# The extracellular matrix of hematopoietic stem cell niches^☆^

**DOI:** 10.1016/j.addr.2021.114069

**Published:** 2022-02

**Authors:** Cornelia Lee-Thedieck, Peter Schertl, Gerd Klein

**Affiliations:** a Institute of Cell Biology and Biophysics, Leibniz University Hannover, Herrenhäuser Str. 2, 30419 Hannover, Germany; b Center for Medical Research, University Medical Clinic, Department II, Waldhörnlestraße 22, 72072 Tübingen, Germany

**Keywords:** Extracellular matrix, Hematopoietic stem cell niche, Bone marrow, ADAM, a disintegrin and metalloproteinase, AML, acute myeloid leukemia, BM, bone marrow, CAMs, cell adhesion molecules, CAM-DR, cell adhesion mediated drug resistance, CAR, CXCL-12 abundant reticular, CFU, colony-forming-unit, CS, chondroitin sulfate, DDR, dimeric discoidin receptor, Del-1, developmentally-regulated endothelial cell locus-1, DG, dystroglycan, DS, dermatan sulfate, ECM, extracellular matrix, EGF, epidermal growth factor, FA, focal adhesion, FAK, focal adhesion kinase, FGF, fibroblast growth factor, FN I - III, fibronectin domain I - III, GAG, glycosaminoglycan, G-CSF, granulocyte colony stimulating factor, GM-CSF, granulocyte macrophage colony stimulating factor, HA, hyaluronic acid, HS, heparan sulfate, HSCs, hematopoietic stem cells, HSPCs, hematopoietic stem and progenitor cells, HSPGs, heparan-sulfated proteoglycans, HxB, hexabrachion, IL, interleukin, KS, keratan sulfate, KLS, c-KIT^+^ Lin^-^ Sca^+^, LM, laminin, LAIR, leukocyte associated immunoglobuline like receptors, LOX, lysyl oxidase, LTR, long-term repopulating, MMPs, metalloproteinases, MSCs, mesenchymal stem/stromal cells, PEG, poly(ethylene glycol), RHAMM, receptor for hyaluronan mediated motility, Robo, roundabout, SCF, stem cell factor, SDF-1 also known as CXCL-12, stromal cell-derived factor 1, SLRPs, small leucine rich proteoglycans, TIMPs, tissue inhibitors of metalloproteinases, trOPN, thrombin-cleaved osteopontin, TLR, toll-like receptor, TGFβ, transforming growth factor β, VCAM, vascular cell adhesion molecule, VEGFR, vascular endothelial growth factor receptors

## Abstract

Hematopoietic stem cells (HSCs) are the life-long source of all types of blood cells. Their function is controlled by their direct microenvironment, the HSC niche in the bone marrow. Although the importance of the extracellular matrix (ECM) in the niche by orchestrating niche architecture and cellular function is widely acknowledged, it is still underexplored. In this review, we provide a comprehensive overview of the ECM in HSC niches. For this purpose, we first briefly outline HSC niche biology and then review the role of the different classes of ECM molecules in the niche one by one and how they are perceived by cells. Matrix remodeling and the emerging importance of biophysics in HSC niche function are discussed. Finally, the application of the current knowledge of ECM in the niche in form of artificial HSC niches for HSC expansion or targeted differentiation as well as drug testing is reviewed.

## Highlights

• Comprehensive overview of different classes of ECM molecules in the HSC niche.
• Overview of current knowledge on role of biophysics of the HSC niche.
• Description of approaches to create artificial stem cell niches for several application.
• Importance of considering ECM in drug development and testing.

## Graphical abstract

**Figure:**
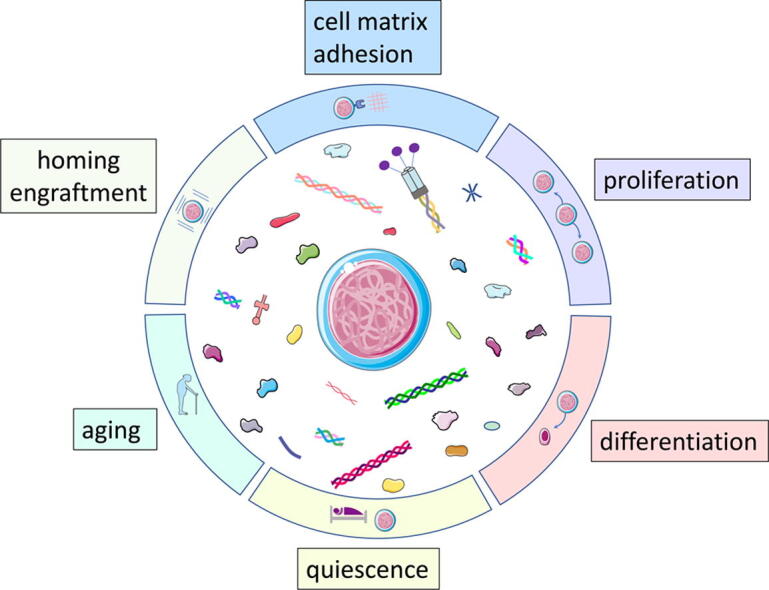

## 1 The hematopoietic stem cell niches: An introduction

Mature blood cells responsible for the defense against pathogens and tumor cells, for wound healing, and for oxygen supply of organs and tissues have to be constantly replaced. Throughout the entire lifetime, multipotent hematopoietic stem cells (HSCs) give rise to all mature blood cells. HSCs represent the apex of the hematopoietic system, but they are not a homogeneous cell population as assumed until recently, but rather characterized by a substantial heterogeneity [1]. In the adult organism HSCs are primarily found in the microenvironment of the bone marrow (BM), representing a protected area. In the late 1970s the British hematologist Raymond Schofield was the first to propose a concept that HSCs are not randomly distributed in the BM microenvironment, but localized in defined areas, the HSC niches [2]. Twenty-five years later first experimental evidence was provided for the existence of an osteoblastic niche at the endosteum [3], [4]. Two years later the existence of a vascular niche was discovered [5], and since that time an intensive research and sometimes controversial discussion was conducted which is the most influential microenvironment for HSCs [6], [7], [8]. Today most researchers agree that HSC niches are perisinusoidal (near the sinusoids), and that the endosteum mainly provides a niche for restricted hematopoietic progenitor cells [9]. The niches control self-renewal, proliferation and differentiation and the trafficking of HSCs and can be viewed as a network consisting of non-hematopoietic or differentiated hematopoietic niche cell types, membrane-bound or secreted signaling molecules of the cytokine or chemokine families and a complex extracellular matrix (ECM) [10]. Despite its prominent occurrence in the BM microenvironment studies about the role of the ECM in the BM niches did not receive a great deal of attention whereas the majority of the “niche literature” focused on the participation of cellular components. There are several excellent reviews about HSC niches where not a single word is mentioned about the functional involvement of the ECM components in the adult stem cell niches [11], [12], [13]. The ECM can provide biophysical and signaling information, and the functions of the ECM comprise the regulation of cell adhesion and migration, control of proliferation and differentiation and determination of cell shape, all important issues in stem cell niches. The purpose of this review is to highlight our current view of the function of the complex ECM in the HSC niches and how this knowledge can be harnessed for drug research.

Towards this end, this review summarizes key issues of BM ECM components in blood cell development. The ECM can be subdivided into several large families. At first we focus on the glycoprotein family, then we discuss the role of different collagen types and finally we highlight the contribution of different proteoglycans to the network of the niches. Elastin as another important ECM molecule is not found in the BM microenvironment and therefore not dealt with in this ECM review. An overview of ECM molecules in the BM and their role in the HSC niches and hematopoiesis is given in Table 1.

**Table 1:** Overview of ECM molecules found in BM and their functions in the HSC niche and hematopoiesis.

ECM component	Influence on HSCs				Influence on hematopoietic maturation			References
	Homing and/or migration	Quiescence and/or self-renewal	Proliferation and/or differentiation	Ageing	B-lymphopoiesis	Erythropoiesis	Myelopoisis	
**Glycoproteins**								
Laminin isoforms	Adhesive for HSPC, enhances homing [14], [15], [16]							Gu et al. 2003 [16], Qian et al. 2006 [14], Qian et al. 2007 [15]

Netrin isoforms				Netrin-1 expression ↓ during ageing [17]			Osteoclastogenesis ↓ [18], [19]	Renders et al. 2021 [17], Mediero et al. 2015 [18], Enoki et al. 2014 [19]

Nidogen					Early B- lymphopoiesis ↑ [20]			Balzano et al. 2019 [20]

Fibronectin	Adhesive for HSPC[21]		Supports an inductive environment for HSPC expansion [22]		Supports maturation of B- cells into plasma cells [23]	FN-dependent erythropoiesis [24]	Supports platelet formation from MK [25]	Dao et al. 1998 [21], Bianco et al. 2019 [22], Nguyen et al. 2018 [23], Eshegi et al. 2007 [24], Malara et al. 2011 [25]

Tenascin isoforms	Adhesive for HSPC [26], [27]		Supports proliferation [27], [28]			Supports erythroid colony formation[29]		Klein et al. 1993 [26], Seiffert et al. 1998 [27], Ohta et al. 1998 [28], Seki et al. 2006 [29]

Fibulins isoforms	Diminishes adhesion of HSPC to FN [30]		Inhibits colony formation of HSPC [30]					Hergeth et al. 2008 [30]

Fibrillins						Restricts differentiation of erythroid progenitors [31]		Smaldone et al. 2015 [31]

Thrombospondins	TSP-1 adhesive for HPC [32], [33], [34]					TSP-4 fragments stimulate erythroid progenitor proliferation [34]		Long and Dixit 1990 [32], Long et al. 1992 [33], Congote et al. 2004 [34]

Osteopontin	Adhesive as a thrombin-cleaved fragment [35]		Suppresses proliferation [36], [37]	Attenuates the ageing process of HSPC [38]				Grassinger et al. 2009 [35], Nilsson et al. 2005 [36], Stier et al. 2005 [37], Guidi et al. 2017 [38]

Osteonectin / SPARC		Quiescence ↓ [39]			Supports B- lymphopoiesis indirectly [40]	Supports erythroid progenitors [41]		Ehninger et al. 2014 [39], Luo et al. 2012 [41], Luo et al. 2014 [40]

Periostin			Proliferation ↓ [42] Proliferation ↑ [43]		Supports B- lymphopoiesis [44]			Khurana et al. 2016 [42], Tanaka et al. 2016 [43], Siewe et al. 2011 [44]

Dermapontin	Adhesive for HPC [45]	Required for *ex vivo* HSC maintenance [46]						Kramer et al. 2017 [45], Kokkaliaris et al. 2016 [46]

Del-1			Induces HSC proliferation [47], [48]				Suports myelopoiesis [47]	Mitroulis et al. 2017 [47], Chen et al. 2018 [48]

Slit 1–3	Effects on HSPC adhesion: Slit-1 ↑ [49] Slit-1 ↓ [50] Slit-2 ↑ [51] Slit-3 ↑ [52]							Smith-Berdan et al. 2011 [49], Goto-Koshino et al. 2012 [50], Waterstrat et al. 2016 [51], Geutskens et al. 2012 [52]

**Collagens**								
Collagen type I		Quiescence ↑ [53]	Proliferation ↓ [53], [54] Proliferation unaffected [55] Proliferation ↑ [56]		Adhesive for B-lymphoid cells [57]	Adhesive for erythroid cells [58]	Adhesive for myeloid cells [58] Pro-platelet formation ↓ [59], [60] Osteoclastogenesis ↓ [61]	Celebi et al. 2011 [53], Oswald et al. 2006 [54], Malara et al. 2014 [55], Choi et al. 2017 [56], Sanderson et al. 1992 [57], Koenigsmann et al. 1992 [58], Semeniak et al. 2016 [59], Balduini et al. 2008 [60], Boraschi-Diaz et al. 2018 [61]

Collagen type III							Pro-platelet formation ↑ [55], [62]	Malara et al. 2014 [55], Fox et al. 2005 [62]

Collagen type IV	Adhesive [63]		Proliferation unaffected [55]				Pro-platelet formation ↑ [59], [60]	Klein 1995 [63], Malara et al. 2014 [55], Semeniak et al. 2016 [59], Balduini et al. 2008 [60]

Collagen type VI	Strongly adhesive [64]							Klein et al. 1995 [64]

Collagen type IX							Myeloid differentiation ↑ Macrophage activation ↑ [65]	Probst et al. 2018 [65]

Collagen type X					B-lymphopoiesis ↑ [66], [67]			Sweeney et al. 2013 [66], Grskovic et al. 2012 [67]

Collagen type XIV					Adhesive for B-lymphoid progenitors [68]		Adhesive for myeloid progenitors [68]	Klein et al. 1998 [68]

Collagen type XVIII							Osteoclastogenesis ↓ [69]	Sipola et al. 2006 [69]

**Proteoglycans**								
Perlecan	Anti-adhesive [70]							Klein et al. 1995 [70]

Agrin			Important for survival and differentiation [71]			Regulates the fitness of the erythroid niche (mouse) [72]	Involved in development and function of monocytes and macrophages [73]	Mazzon et al. 2011 [71], Anselmo et al. 2016 [72], Mazzon et al. 2012 [73]

Small leucine-rich proteoglycans		Biglycan is higher expressed in LT-HSCs compared to ST-HSCs [74]	Decorin regulates indirectly the number and lineage preference of HSCs [75] Biglycan stimulates differentiation of monocytic cells from HSC [76]		Decorin indirectly blocks B-lymphopoiesis [75]			Forsberg et al. 2005 [74], Ichii et al. 2012 [75], Tomoyasu et al. 1998 [76]

Syndecan					Mediates B- lymphocyte interactions with matrix [57]		Enhances motility of macrophages ↑ [77]	Sanderson et al. 1992 [57], Angsana et al. 2015 [77]

Glypican	Enhances homing ↑ [78]						Involved in hematopoiesis and important for osteoclastogenesis (mouse) [79]	Khurana et al. 2013 [78], Viviano et al. 2005 [79]

**Hyaluronan (HA)**								

Hyaluronan	Involved in HSPC mobilization [80] Low molecular weight HA decreases HSPC migration towards SDF-1 [81] Participates in HSPC lodgment at the endosteum following transplantation [82] Important for migration of transplanted HSPC into the marrow [83]		Involved in the regulation of HSPC proliferation and differentiation [82] Addition of exogenous HA enhances HSPC proliferation [84]				Enhances/facilitates hematopoiesis [85], [86] Involved in thrombopoiesis [87] Low molecular weight HA polymers mobilize leukocytes [81] Triggers M2-like polarity of monocytes/macrophages [88]	Pilarski et al. 1999 [80], Schraufstatter et al. 2009 [81], Nilsson et al. 2003 [82], Goncharova et al. 2012 [83], Lee-Sayer et al. 2018 [84], Khaldoyanidi et al. 1999 [85], Maztrosova et al. 2004 [86], Petrey et al. 2016 [87], Kim et al. 2019 [88]

To complete the picture of HSC niches provided in this review, we will precede the intense discussion of the ECM with a brief overview of the cellular components of HSC niches.

## 2 Cellular complexity in the hematopoietic stem cell niches

Most of our knowledge about the cellular constituents of BM HSC niches comes from studies in mice [13]. A “niche cell” supporting HSC stemness and maintenance is primarily identified by calculating the distance between a HSC and a particular cell type under study given the fact that a cell in close proximity is more likely to determine the fate of the HSC than a distant cell [89]. This definition critically depends on the unambiguous identification of HSCs in the intact tissue. For fluorescence-based 2D-confocal microscopy this requirement is hampered by the rarity of HSCs in the tissue and the limited number of markers which can be applied in studies with immunostained sections [5], [90], [91], [92]. In recent years a significant step forward came with the introduction of sophisticated 3D-volumetric multicolor imaging methods [93], [94], [95], [96]. Here major improvements were obtained by applying tissue clearing protocols which allowed deeper imaging depths [97] and newly introduced reporter mouse models with specifically-labelled HSC subsets [98], [99], [100], [101]. The insights obtained with the imaging analyses were often corroborated employing transgenic mouse models where different endogenous BM cell types or niche factors were ablated [3], [4], [92], [102], [103], [104], [105]. However, for some genetically deleted cell types it is still not clear whether the observed functional consequences of the deletion for HSCs were direct or indirect. Rather new approaches to functionally define niche cell candidates in the BM are the use of mass cytometry-based single-cell analysis [106] and the combination of single-cell and spatially resolved transcriptomics [107].

The diversity of niche cells identified by the different aforementioned approaches ranges from mesenchymal stromal cells to mature hematopoietic cells leading to the paradoxical situation that almost all cellular components in the BM microenvironment may have a more or less important function for the control of HSCs [12]. The cells identified as candidate niche cells mainly include endothelial cells of the sinusoids and arterioles [5], [108], [109], [110], [111], [112], [113], [114], [115] and perivascular cells such CXCL-12 abundant reticular (CAR) cells or nestin^+^ or leptin-receptor^+^ mesenchymal stromal cells [92], [108], [116], [117], [118]. Other cells in the BM microenvironment with a reported association to HSCs are osteolineage cells such as BM mesenchymal stem/stromal cells (MSCs; including skeletal stem cells), osteoblasts and even osteocytes [3], [119], [120], [121], adipocytes [105], [122], cells of hematopoietic origin such as megakaryocytes [102], [123], [124], osteomacs and osteoclasts [104], [125], and neuronal cells such as nonmyelinating Schwann cells [126], [126], [127]. With a plethora of reports on different BM niche cells, sometimes contradictory results might be explained by the use of different methods employed, the study of transplanted vs. homeostatic HSCs in the BM or the analysis of different bone tissues such as tibia, femur, sternum or calvarium [94].

Numerous BM niche cells can produce and secret key HSC regulators such as SDF-1 (stromal cell-derived factor 1, also known as CXCL-12), angiopoietin 1 or stem cell factor (SCF) (reviewed in [128]), but the relative contribution of the different niche cell populations as an essential source of these cytokines is still not fully understood, since the expression of the different factors is not restricted to individual niche cell types. Beside these cytokines, the different BM niche cells also produce ECM components which build up matrices of various stiffness in the BM microenvironment. Osteoblasts are involved in bone formation, a tissue of very high stiffness [129]. The different MSC types can synthesize a very soft interstitial matrix which can be detected as specialized reticular fibers in between the sinusoidal network [130]. And endothelial cells, adipocytes and neuronal cells can synthesize basement membranes, a structured matrix of intermediate stiffness [131]. Since HSCs and their more differentiated progenitors can sense biomechanical signals [132], the spatial localization of these cells along with their embedding in a soft or stiff matrix can certainly influence their fate.

Most of the studies in mice clearly favored the role of vascular niches and CAR cells for HSC maintenance [12], [13], [133], [134], [135]. And although many aspects of HSC niches are assumed to be quite similar between mice and humans several studies reported differences between human and murine niches indicating that results obtained in murine niches may not always be comparable to humans (examples are found in [136], [137]). An integrated model of a (murine and human) hematopoietic niche which takes the specific contributions of all suggested niche cells into account is currently not available, and therefore there is still a range of open questions: (1) is the localization of the HSC in their niches actively selected or does it depend on the relative abundance of the BM niche cells [8] ?; (2) which niche cell types are mandatory to build up an artificial (human) stem cell niche?; and (3) is our already complex current view of the different contributing cell types to hematopoietic niches still too simplistic ?

## 3 ECM glycoproteins in the bone marrow

**Fig. 1:**
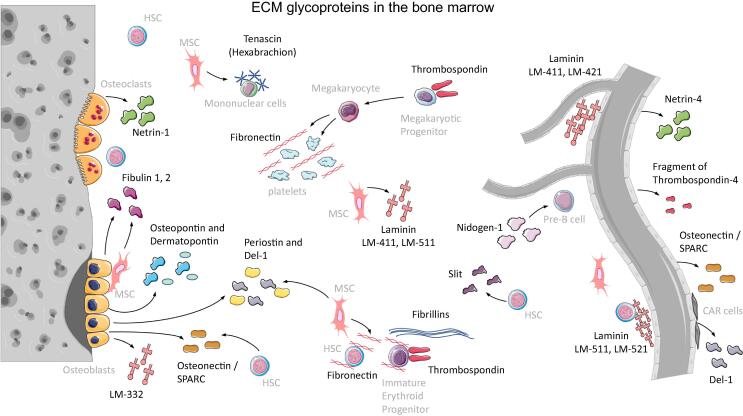
ECM glycoproteins in the bone marrow. The localization of the most important glycoproteins within the HSC niche are shown and which cells in the HSC niche are able to express these glycoproteins. The endosteal niche is shown on the left and the vascular niche on the right. Figure was created with the help of Servier Medical Art by Servier, licensed under a Creative Commons Attribution 3.0 Unported License.

Glycoproteins are a family of glycosylated multifunctional proteins that exert a vast variety of functions in the BM as discussed below in detail. The glycoproteins playing a role in the BM HSC niche are illustrated in Fig. 1 and a summary of their functions is included in Table 1.

### 3.1 Laminin isoforms

Laminins are a family of large heterotrimeric molecules consisting each of an α, β and a γ chain. Five α (α1-α5), three β (β1- β3) and three γ (γ1- γ3) chains have been identified and characterized which give rise to at least sixteen different isoforms with different biological functions [138], [139], [140]. The nomenclature of the laminins reflects the chain composition of the individual isoforms. LM-521, as an example, consists of the α5, the β2 and the γ1 chain [141]. Laminins are major components of all basement membranes that underlie epithelial or endothelial cells or surround adipocytes, nerve fibers or muscle cells [142]. In the BM, basement membranes are located around sinusoids and larger arterioles, but also around nerve fibers and fat cells. The major laminin isoforms in the BM are those containing an α4 or α5 chain, whereas isoforms containing an α1 chain are not expressed [143], [144]. The α2 chain is only found in large blood vessels in humans [143], and in murine BM this chain can only be detected in basement membranes wrapping nerve fibers [130]. Signals for the α3 chain can be detected in larger blood vessels [130], [145], but not in human BM stromal cells which have been reported to synthesize an unusual LM-522 isoform not yet detected in other human tissues [145]. LM-511 and LM-521 are major components of sinusoidal and arteriolar basement membranes, whereas laminin isoforms containing the α4 chain (LM-411, LM-421) are not only found in these endothelial basement membranes, but also in an intricate intersinusoidal fiber network, both observed in human and in mouse BM [130], [143]. Although this network is reminiscent of the conduit network of secondary lymphoid organs, its dimension and composition suggest that it is structurally different [130], [146]. A deletion of the α4 chain in laminin α4-deficient mice led to a reduced proliferation and impaired recirculation of migratory hematopoietic stem and progenitor cells (HSPCs) which might be explained by a compensatory expression of α5 chain-containing laminins [130]. Cell adhesion assays revealed that α5 chain-containing isoforms are strong adhesive substrates for human and mouse HSPC [16], [143], [147] and also for a variety of erythroid, myeloid and lymphoid cell lines.

Of the three laminin β chains the most prominent expression pattern was found for the β2 chain [130], [143], [148]. The laminin β1 chain could only be detected in human, but not in mouse BM, and the β3 chain was lacking in both organisms [130], [143]. Of the three γ chains a prominent expression of the γ1 and γ2 chains was observed both in mouse and human BM tissue and BM stromal cells, whereas an expression of the γ3 chain could not be detected [130], [145].

Although an expression pattern of the α4 chain or α5 chain-containing isoforms has been found in granulocytes, lymphoid and monocytic cells and in platelets and megakaryocytes, hematopoietic lineage-negative progenitor cells do not seem to synthesize laminin α chains [130], [149], [150], [151], [152], [153]. This is in contrast to the second multipotent stem cell type of the BM, the MSCs. Although MSCs do not deposit a structured basement membrane, human BM MSCs significantly synthesize LM-411 and LM-511 [154]. During adipogenic differentiation of MSCs the expression of LM-411 is substantially enhanced [155]. Whether the secreted, MSC-derived isoforms are required for the generation of the intersinusoidal reticular meshwork is an open question.

### 3.2 Netrins and nidogens

Netrins are a family of laminin-related, secreted proteins which can regulate divers processes such as adhesion, migration, proliferation and differentiation [156]. In the BM, netrin-1 plays a major role in regulating HSC dormancy and self-renewal. This influence of netrin-1 is mediated through the cell surface receptor neogenin-1 mainly expressed by dormant HSCs [17]. The main source of netrin-1 in the BM are endothelial and perivascular cells, but during ageing a decline of netrin-1 expression by these cells can be observed. This loss of netrin-1 synthesis leads to a compensatory upregulation of neogenin-1 on aged HSCs that, however, is not sufficient to control HSC quiescence [17].

The bone-resorbing osteoclasts can participate in stem cell niche maintenance [157]. Netrin-1 is also a paracrine factor produced by osteoclast precursors that can regulate the differentiation of these cells into more mature osteoclasts [18]. On the other hand, netrin-4 synthesized by vascular endothelial cells can inhibit the differentiation process of osteoclasts [19]. A similar inhibition of osteoclastogenesis was observed for LM-332 which was found to be expressed in murine osteoblasts [158].

Nidogen-1, together with nidogen-2, are essential components of all basement membranes bridging the laminin network with the collagen type IV network [142]. Although expression and function of laminins and collagen type IV in the BM have been well documented, astonishingly very little information is available for both nidogens in the hematopoietic microenvironment. Pre-B-cells which seem to share a common perivascular niche with HSCs are retained in their niche by an interaction with nidogen-1. Loss of nidogen-1 in peri-sinusoidal stromal cells impaired the differentiation of early B-lymphocytes [20]. This work provided strong evidence that multi-specific niches can co-exist supporting both stem and more differentiated progenitor cells.

### 3.3 Fibronectins

Fibronectin exists as a soluble molecule in the blood plasma, but as an insoluble, deposited matrix molecule it is also a major structural component of the BM [159], [160]. Fibronectin is a homodimer consisting of two homologous chains linked by disulfide bridges. A variety of fibronectin isoforms have been identified which arise from alternative splicing of a single gene [161], [162]. The individual fibronectin chains can be subdivided into defined repeating protein domains designated fibronectin type I, type II or type III domains (abbreviated FN I, FN II, FN III). Interactions with more than ten different integrin receptors have been mapped to individual FN III domains. Beside these binding sites for integrins there are also binding sites for heparin, chondroitin sulfate (CS) proteoglycans, and collagens or gelatin which can give rise to larger ECM complexes [163].

A major role for fibronectin in the BM microenvironment is the regulation of developing erythroid cells [164]. After an early erythropoietin-dependent, fibronectin-independent stage, a stage dependent on adhesive interactions with fibronectin follows [24]. The immature erythroid progenitors strongly attach to fibronectin, but at late maturation stages of erythroid development adhesion to fibronectin is drastically decreased indicating an inverse correlation of adhesion and erythroid maturation [165]. For early erythroid progenitors fibronectin also seems to be a growth-promoting factor mediated mainly through the integrin α4β1 [166], [167]. However, conflicting data were also reported suggesting fibronectin as an inhibitor of erythroid formation [168]. Recently, fibronectin has been identified as an essential factor of the BM supporting the maturation of migrating antibody-secreting B-cells into stationary long-lived plasma cells in the BM [23]. A direct involvement of fibronectin in the BM has also been detected for megakaryocyte differentiation and release of platelets [25]. Here, fibronectin is directly secreted by megakaryocytes building an integral part of the pericellular matrix surrounding these platelet-budding cells [55]. Another major source of fibronectin in the BM are MSCs [169]. When human BM MSCs were used to colonize a porous hydroxyapatite scaffold giving rise to a biomimetic HSC niche, a prominent deposition of fibronectin could be observed [170]. Fibronectin is also found to be a major extracellular component of a decellularized BM bio-scaffold used as an inductive microenvironment for HSC expansion [22]. Synthesis and secretion of fibronectin by BM stromal cells can be regulated by glucocorticoids, e.g. dexamethasone that rapidly down-regulates fibronectin expression [171].

### 3.4 Tenascins

Of the four tenascin family members (tenascin-C, -R, -W and –X), only tenascin-C is highly expressed in the healthy BM microenvironment [26], [172]. A prominent expression of tenascin-W in the BM is only induced when the hematopoietic microenvironment serves as a metastatic niche for circulating tumor cells [173]. Tenascin-C, the best-studied member of the tenascin family, is widely expressed in developing tissues, but in the adult organism its expression is restricted to highly regenerative tissues such as the hematopoietic system within the BM. This matricellular molecule consists of six identical subunits assembled at their N-terminal ends in a structure known as ‘hexabrachion’ (HxB) [174]. Each subunit contains epidermal growth factor (EGF) and FN III repeats, followed by a C-terminal fibrinogen homology domain. Due to alternative splicing, several defined isoforms of tenascin-C can be generated, but in human or murine BM only two major isoforms have been detected, a larger form (HxB.L) containing the FN III repeats TNfnA-D and a smaller form (HxB.S) lacking these FN III domains [26], [27], [172]. Glucocorticoids have been shown to down-regulate tenascin-C expression in long term BM cultures, primarily affecting the larger splice variant [172], [175].

Several experimental data sets revealed that tenascin-C can exhibit anti-adhesive effects [176], however for BM mononuclear cells and various myeloid cell lines tenascin–C seems to be a strong adhesive substrate [26], [27]. These adhesive interactions were shown to be mediated by specific domains of the FN III repeats and the C-terminal fibrinogen-like knob. Heparin, but not function-blocking antibodies against integrin chains, could inhibit these interactions indicating that membrane-bound proteoglycans are the responsible cellular receptors [27]. Multiple myeloma cells, however, attached only weakly to tenascin-C, although this protein was prominently expressed in the BM of multiple myeloma patients [177].

Tenascin-C deficient mice have been reported to develop normally, and their HSC pool in the BM is not altered [178], [179]. Nevertheless, hematopoietic cell production is substantially repressed in long term BM culture of tenascin-C deficient mice [28]. These results are in line with the capacity of purified tenascin-C preparations to strongly stimulate proliferation of BM mononuclear cells [27]. Concomitantly with hematopoietic cell recovery after myeloablation, a dramatic up-regulation of tenascin-C expression can be observed indicating a substantial role for tenascin-C in hematopoietic recovery [180]. An analysis of different BM stromal cell lines supporting erythropoiesis identified tenascin-C as a key molecule for stromal-dependent erythroid development [29]. Taken together these reports indicate that tenascin-C has a relevant function in normal hematopoietic cell development.

### 3.5 Fibulins, fibrillins and thrombospondins

Fibulins are a family consisting of seven members (fibulin-1 to fibulin-7) each characterized by a typical C-terminal fibulin-type structural motif [181], [182]. Their main function consists in cell-to-matrix communication, but they also have binding sites for other ECM molecules leading to larger protein complexes. In the BM the isoforms fibulin-1 and fibulin-2 isoforms are prominently expressed [30], [183]. Osteoblasts are a rich source of both fibulins [30] and both isoforms are also expressed by BM stromal cells where the secreted fibulins are found deposited in close association with fibronectin [183]. As for fibronectin, the expression of both fibulins can be suppressed by glucocorticoids [171]. Fibulin-1 and -2 are no adhesive substrates for human CD34^+^ HSPCs, on the contrary, fibulin-1 can drastically diminish the strong adhesion of CD34^+^ cells to fibronectin thus regulating the overall adhesion strength in the BM microenvironment [30]. An influence on hematopoietic progenitor cell proliferation was observed in colony formation of erythroid and myeloid cells which was diminished by the addition of fibulin-1 or fibulin-2 [30].

Fibrillin-1 and -2 are major structural components of microfibrils, which are only very low abundance structures in the BM [184]. Nevertheless, fibrillin-1 can be found deposited in erythroblastic niches in the marrow microenvironment. Loss of fibrillin-1 in fibrillin-1-deficient mice leads to an increased number of mature erythrocytes indicating that fibrillin-1 can restrict the differentiation of developing erythroid progenitors [31]. Although myeloid differentiation was not affected in number and potency in fibrillin-1 null mice, a decreased frequency of HSCs was noted in these animals [31]. These results implicate that fibrillin-1 differentially regulates stem and progenitor cells in early stem cell niches and in more mature erythroid niches.

In humans and mice, the thrombospondins comprise a family of secreted homotrimeric or homopentameric glycoproteins with five members (thrombospondin-1 to thrombospondin-5) [185]. Thrombospondin-1 seems to be the most prominent member of this family in the BM, very low expression levels for thrombospondin-2 and -3 were found in human BM [186]. Human hematopoietic progenitor cells of the erythroid, megakaryocytic and myeloid lineages strongly attach to thrombospondin-1, but during further maturation attachment to thrombospondin-1 gradually decreases [32], [33]. The adhesive interactions of the early progenitors mainly seem mediated by membrane-bound heparan sulfate (HS) proteoglycans, and not by RGD-dependent integrins, although thrombospondin-1 contains an RGD motif in each of its subunits [32]. A heparin derivative was also found to counteract an inhibitory function of thrombospondin-1 on the growth of megakaryocytic colonies *in vitro* [187]. This inhibitory role was at least in part due to the binding of thrombospondin-1 with CD36 expressed on megakaryocytic progenitors [188]. Conversely, a fragment of thrombospondin-4 generated by endothelial cells under the influence of erythropoietin can stimulate the proliferation of CD36^+^ erythroid progenitors [34] indicating that the thrombospondin family can differentially modulate erythroid and megakaryocytic progenitors in the BM.

### 3.6 Osteopontin

Osteopontin is a highly acidic secreted matrix protein of the SIBLING (**s**mall **i**ntegrin-**b**inding **li**gand **N**-linked **g**lycoprotein) family. In the BM, osteopontin is preferentially expressed by bone-lining osteoblasts. Two publications in 2005 highlighted that osteopontin can suppress the proliferation of murine HSC thus regulating the stem cell pool size [36], [37]. As an adhesive substrate, osteopontin can also influence the lodgment of HSPCs in the BM after stem cell transplantation. This is mainly achieved by the thrombin-cleaved osteopontin (trOPN) fragment, the prevalent form of osteopontin in the murine and human BM [35]. The proteolytically-generated trOPN fragment releases a new binding site for the integrin α9β1, which is strongly expressed on human and murine HSPCs [35], [189]. Recently it was found that ageing effects on HSCs can also be mediated by osteopontin. Upon ageing, a decreased expression of osteopontin can be observed in murine BM microenvironment. When aged murine HSPCs were treated with trOPN, the ageing process of HSPCs was attenuated leading to a better engraftment of HSPCs and a new balance of myeloid and lymphoid cells in the periphery [38]. This balance is regulated by the secreted trOPN isoform, but also by a second isoform found intracellularly. Whereas the secreted form can increase the size of the lymphoid cell population, the intracellular isoform can diminish the number of myeloid progenitors and more mature myeloid cells [190]. Thus, an unbalanced ratio of secreted and intracellular osteopontin can skew the balance of myeloid and lymphoid cells in the blood.

### 3.7 Osteonectin / SPARC

The matricellular protein osteonectin is a 40 kDa acidic and cysteine-rich glycoprotein hence also synonymously called SPARC which stands for ‘*secreted protein acidic and rich in cysteine’* [191]. Osteonectin is highly expressed by osteoblast and by BM endothelial cells in the BM microenvironment, where it can support the development of erythroid progenitor cells as shown by the exogenous addition of osteonectin to human CD34^+^ cells [41]. Osteonectin expression was found to be upregulated in HSPCs that have migrated from the fetal liver and colonized the BM after birth. This autocrine expression of osteonectin, however, does not seem to be responsible for their homing and engraftment [39]. Osteonectin can regulate the proliferation of HSCs since its loss in osteonectin-deficient mice leads to an accelerated return of HSCs to a quiescent state after cytotoxic treatment thus protecting HSCs from lethal effects of chemotherapy [39]. Osteonectin-null mice also showed an impaired B-lymphopoiesis. Conditioned medium from osteonectin-deficient BM stromal cells could inhibit B-cell differentiation *in vitro*. However, when BM progenitors were cultured on osteonectin-null stromal cells, the addition of recombinant osteonectin did not increase the number of newly formed B-cells indicating an indirect effect of the matrix protein [40].

Osteonectin also plays a role in various pathological conditions. In B-cell progenitor acute lymphoblastic leukemia the tumor cells strongly adhere to BM stromal cells. The proteasome inhibitor bortezomib strongly increases osteonectin expression in the stromal cells which leads to a drastic decrease in tumor cell attachment to the stromal cells [192]. A drastic decrease in osteonectin expression by BM stromal cells is observed in aplastic anemia with hypocellularity in the BM of the patients [193]. Using a prostate BM metastasis model Sharma and coworkers found that the expression of osteonectin by tumor cells can up-regulate bone morphogenetic protein 7 secretion by stromal cells leading to quiescence of the tumor cells [194]. Thus, osteonectin in the BM microenvironment affects proliferation/quiescence and adhesion of both hematopoietic progenitors and metastasized tumor cells.

### 3.8 Periostin

The matrix molecule periostin, originally identified in an osteoblastic cell line, belongs to the small fasciclin I family [195]. Interestingly, periostin is one of only 12 proteins in humans in which the glutamic acid residues are modified to γ-carboxyglutamic acid in a vitamin K dependent mechanism introducing a higher affinity for calcium ions [196]. In the BM, periostin is mainly expressed by osteoblasts and their progenitors, the MSCs. The early B-cell factor was identified as the responsible transcription factor for the expression of periostin in BM stromal cells [197]. Periostin seems to play an important role in B-lymphopoiesis as decline of periostin expression dramatically affects B-cell development [44]. In line with these results periostin also promotes B-cell acute lymphoblastic leukemia progression [198]. Periostin also has an influence on the proliferation of HSCs, but here non-overlapping results were published. Whereas Khurana et al. reported that recombinant periostin can inhibit proliferation of KLS (c-KIT^+^ Lin^-^ Sca1^+^) cells cultured in the presence of SCF and thrombopoietin, Tanaka and coworkers showed that the number of HSPCs including long-term culture-initiating cells were enhanced by the addition of periostin to a specific co-culture of murine BM cells with stromal cell types [42], [43]. Interestingly, in both reports the effect of recombinant periostin was mediated through the integrin αvβ3. Whether the different results were due to the different experimental approaches has still to be clarified. Since periostin is often found to be highly expressed in tumor microenvironments [199], expression of periostin in the adult BM can help to establish a pre-metastatic niche for tumor cells with a predilection for metastasis formation in the BM, such as breast or prostate cancer cells.

### 3.9 Del-1, dermapontin, Slit-3

***D****evelopmentally-regulated****e****ndothelial cell****l****ocus-1* (Del-1) is a secreted 52 kDa ECM protein consisting of three EGF-like repeats and two discoidin-I-like domains [200]. Its expression was originally thought to be restricted to embryonic vascular tissues [201], but in the adult BM Del-1 was also found to be prominently expressed in different cellular components of the HSC niches, including osteoblasts, MSCs, CAR cells and endothelial cells [47]. Del-1 can regulate long-term HSC proliferation and the differentiation of hematopoietic progenitors towards myelopoiesis [47], [48]. This is mediated by an interaction of integrin αvβ3 expressed by the HSPCs with an RGD motif in the second EGF domain of Del-1. For a successful engraftment of transplanted stem cells in the recipient BM, the expression of Del-1 is required [47]. It is likely that the inhibition of Del-1 in the BM microenvironment can enhance the mobilization of HSCs into the periphery.

A small 24 kDa non-collagenous matrix molecule with a strong influence on HSPC proliferation is dermatopontin, also known as TRAMP (**t**yrosine-**r**ich **a**cidic **m**atrix **p**rotein) [202]. In the BM, dermatopontin is mainly expressed by osteoblasts, (see: http://biogps.org/#goto=genereport&id=56429). Analyzing the supportive activity of ATF024 cells for HSCs, Kokkoliaris and coworkers found that dermatopontin is elementary for the *ex vivo* survival and expansion of HSCs with a long term engraftment capacity [46]. Dermatopontin can also promote integrin-mediated adhesion of hematopoietic progenitors, but does not seem to be necessary for steady-state hematopoiesis *in vivo* [45].

The three members of the highly homologous Slit family (Slit-1, Slit-2, Slit-3) are large matrix molecules known for their inhibitory role in leukocyte and neuronal cell migration [203]. Slits are recognized by receptors of the **Ro**unda**bo**ut family which consists of four members (Robo1-4) [204]. In the BM microenvironment transcription of all three Slits could be detected, and Robo1 seems to be the main receptor for Slits expressed by human CD34^+^ HSPCs [52], [205]. On the contrary, murine KLS long-term repopulating (LTR) HSCs also express Robo4 [206]. Using a Robo4-deficient mouse model Smith-Berdan and coworkers stated that Robo4 played a functional role in the retention of LTR stem cells in the BM microenvironment [49], whereas Goto-Koshino *et al.* reported contradictory results [50]. Ectopic expression of Slit-2, the ligand for Robo4, could increase the amount of long-term colony forming HSCs and also their engrafting capacity after stem cell transplantation [51]. Pre-treatment of human CD34^+^ HSPCs with Slit-3 could inhibit their *in vitro* migratory activity. However, *in vivo*, this pre-treatment increased their homing efficiency to the BM, without an influence on the proliferation of the hematopoietic progenitors [52]. Together, these results point to an essential role for Slits in the BM microenvironment.

## 4 Collagens in the bone marrow

### 4.1 General

In mammals, collagens account for approximately 30% of the total mass of all proteins and are thus the most abundantly expressed protein class. They are a large protein family with 28 members that share at minimum one triple helical domain as a common structural feature. Each collagen is composed of 3 polypeptide chains called alpha chains that can form homo- or heterotrimers. Within each triple helical domain, the alpha chains are screwed in a right-handed triple helix [207]. The tight contact between the polypeptide chains along the central axis of the triple helix is enabled by the presence of the small amino acid glycine in every third position, leading to glycine-X-Y repeats, where X and Y are often proline or hydroxy-proline. The glycine residues are located in the center of the helix [208]. Collagens are mostly found as supramolecular assemblies within the ECM of tissues [207]. Depending on their structure and structure of their assemblies, collagens can be subdivided into fibril forming collagens (collagen type I, II, III, V, XI, XXIV, and XXVII), fibril-associated collagens with interrupted triple helices (FACIT; collagen type IX, XII, XIV, XVI, and XIX to XXII), network-forming collagens (collagens type IV, VIII, and X), membrane-bound collagens (collagen type XIII, XVII, XXIII, XXV), multiplexins (collagens type XV and XVIII) and other collagens (collagen type VI, VII, XXVI, and XXVIII) [207]. Members of each of these subgroups except membrane-bound collagens were described to be expressed in BM and they play roles in various hematopoietic processes, as outlined below. Collagens have structural and mechanical functions. They contribute to the organization and shape of tissues as well as tissue integrity [207]. Cells can interact with collagens via specific receptors, and in this way collagens also function in the regulation of cell adhesion, migration, proliferation or differentiation. Receptors for collagens include integrins, dimeric discoidin receptors (DDR), glycoprotein VI and leukocyte-associated immunoglobulin-like receptors (LAIR) [209]. Besides the function of the entire, intact collagen molecules, proteolytic degradation products of collagens can also confer biological activity that differs from that of their parent molecules. These collagen fragments — mainly evolved from molecules of the basement membrane — are termed ‘matricryptins’. They are involved in the regulation of physiological processes such as angiogenesis, development and tissue repair or in pathological processes such as tumor growth and metastasis. Therefore, they are potential drugs. The best-known example is endostatin, the C-terminal fragment of collagen type XVIII [207], [210].

Due to their structural properties as well as their inherent biocompatibility and biodegradability, collagens are used in drug delivery as vehicles and biomaterials (e.g. gelatin capsules or growth factor-releasing hydrogels). Besides, collagens can also be used as sites for drug targeting due to changed expression patterns in diseases like fibrosis [208].

**Fig. 2:**
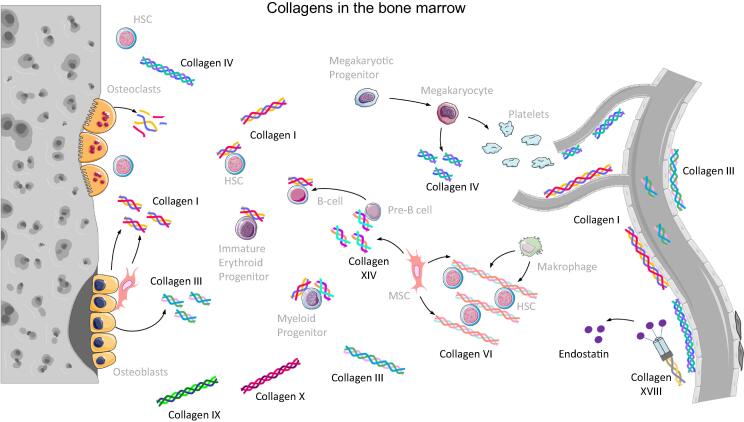
Collagens in the bone marrow. Cells which are able to express the most important collagen types are shown within the HSC niche. On the right side the vasculature within the bone marrow is depicted forming the vascular niche of HSCs. On the left side bone structure is shown resembling the endosteal niche. Figure was created with the help of Servier Medical Art by Servier, licensed under a Creative Commons Attribution 3.0 Unported License.

Here we will focus on collagens that are present in BM and for which a function in the HSC niche or hematopoiesis has been described (Fig. 2, Table 1).

### 4.2 Overview of collagens in BM

Currently, there exists no comprehensive overview of collagens expressed in BM. In the 1990s expression of collagens type I, III, IV, V, VI and XIV in the BM was shown on the protein level [63], [64], [68], [211], [212]. On the mRNA level, collagen type II was additionally described [63]. Since then, also collagens type IX, X and XVIII were connected to the hematopoietic environment [65], [66], [69], [213], [214]. Collagens type II and XI were demonstrated to play a role in the survival niches of memory CD4^+^ T lymphocytes in BM [215], and collagen type XV was shown to be expressed by human MSC in a hypoxia-regulated manner [216], [217]. In the following, all collagens for which a role in the hematopoietic microenvironment is well-established will be discussed one by one. They are also illustrated in Fig. 2 together with cells expressing them.

### 4.3 Collagen type I

Collagen type I is the most abundant collagen family member and is found ubiquitously in many tissues. Large amounts of this collagen are deposited in the bone matrix [211], where it can be detected in compact and trabecular bone [212]. In BM, collagen type I is mainly expressed by osteoblasts [218] but also by bone marrow stromal cells [219], [220] including MSCs [221]. Its synthesis is regulated by several factors and cytokines. Amongst them, TGF β1 takes a central role and triggers the upregulation of collagen type I expression in human MSCs by repressing sphingosine-1-phosphate receptors. Of note, these effects are different in murine MSCs *in vitro* [222]. Reports on the distribution of collagen type I in the BM cavity are quite divers, probably due to species-related differences between mice, rats and men or due to differences in decalcification protocols applied prior to staining. In mice, collagen type I was reported to be located at the endosteum and no expression was detected in the central marrow region or the marrow vessels in this region [212]. Newer studies report collagen type I to be present in thin filaments or fibers throughout the BM cavity and around larger arteries and arterioles [55], [59]. In human and rat BM, collagen type I expression was found in a fibrillary network and particularly strong staining was observed around several blood vessel types [223], [224].

Collagen type I was described to be an adhesive substrate for erythroid and myeloid progenitor cells [58]. At the same time many hematopoietic cell lines that represent hematopoietic cells during different maturation stages cannot adhere to collagen type I *in vitro* [64]. It was shown that B-lymphoid and myeloma plasma cells recognize collagen type I via the receptor syndecan 1 [57], [63], [225]. 20% of freshly isolated CD34^+^ HSPCs from umbilical cord blood express integrin α2β1 as a collagen type I receptor and this percentage increases during *in vitro* culture and the concomitant myeloid differentiation. Culturing HSPCs on surfaces coated with collagen type I fibrils results in diminished proliferation and altered differentiation. These observations led to the hypothesis that collagen type I might contribute to the quiescence of HSCs in the endosteal niche [53]. This is supported by the finding that frequencies of colony forming units in HSPC cultures on collagen type I gels are increased at diminished total expansion [54], while others report that collagen type I does neither increase viability nor expansion of cultured KLS cells [55] or that KLS cell proliferation is higher on collagen type I-coated hydrogels in comparison to LM-111- or fibronectin-coated hydrogels [56].

In hematopoietic differentiation collagen type I plays a role in platelet formation and osteoclastogenesis. Proplatelet formation is inhibited by collagen type I via glycoprotein VI receptors [59], [60]. Similarly, osteoclastogenesis from hematopoietic progenitors is inhibited by collagen type I and its degradation products via the receptor LAIR-1. Interestingly, immobilized collagen type I had only low inhibitory activity. These findings indicate that collagen type I digestion by mature osteoclasts during bone resorption provides a negative feedback loop limiting osteoclastogenesis via the released bioactive collagen type I fragments [61].

### 4.4 Collagen type III

Collagen type III is mainly secreted in hollow and extensible tissues such as blood vessels, bowel and uterus [226]; small amounts are found in bone where it is secreted by osteoblasts [227]. Similar to collagen type I, reports on the expression pattern of collagen type III in bone and BM are heterogeneous. Some authors report that collagen type III is absent from cortical bone [59], [212] whereas others find it in the bone structure [55]. In the marrow, collagen type III was described to be found throughout the marrow [59] or as few fibrils in marrow and around arterioles [55] or in periostal regions but absent from endosteal regions and central marrow including vessel structures [212]. Functionally, collagen type III seems to be involved in the development of trabecular bone by affecting osteoblastogenesis [227] and proplatelet formation [55]. For hematopoietic cell lines, collagen type III was described to be a non-adhesive substrate [64].

### 4.5 Collagen type IV

Generally, collagen type IV is predominantly deposited in basement membranes [63]. In the BM, collagen type IV is found in the endosteal region as well as the central marrow around BM vessels including sinusoids [55], [59], [212]. Collagen type IV proved to be an adhesive substrate for some hematopoietic cell lines, however, it is not adhesive for hematopoietic progenitor cells [63]. Accordingly, collagen type IV does not improve KLS cell multiplication and survival [55]. During hematopoietic differentiation collagen type IV supports proplatelet formation at sinusoids [60], where it overrides the inhibitory effects of collagen type I by stronger cell binding [59]. Megakaryocytes were shown to express collagen type IV [55] and thus their role in the hematopoietic system in BM is not limited to platelet formation by releasing them from their cell body; they also contribute to the composition of the ECM microenvironment in vascular niches. This assumption is underpinned by the finding that collagen type IV expression is upregulated during stressed hematopoiesis, when platelet counts are strongly decreased [55].

### 4.6 Collagen type VI

Collagen type VI forms microfibrillar structures. In human BM it is found in extrasinusoidal spaces in between developing hematopoietic cells. Hematopoietic cell lines adhere strongly to collagen type VI and this interaction is at least partially mediated by syndecan receptors [64]. Treatment with granulocyte colony stimulating factor (G-CSF) can lead to a reduction of expression and secretion of collagen type VI by MSCs. Therefore, it appears possible that the interaction of HSPCs with their niche is partially governed by the presence of collagen type VI in this microenvironment and its regulation by G-CSF [228]. Furthermore, another important cell type in the HSC niche, the macrophages, were shown to express collagen type VI abundantly and to use this molecule to modulate their cell binding properties [229]. Moreover, multiple myeloma cells were shown to be able to bind to collagen type VI. Collagen type VI is expressed in the BM of patients suffering from multiple myeloma in a pattern that is not different from the one found in samples from healthy individuals. While a role for integrins could be excluded for this interaction, the responsible cellular receptors remain to be elucidated [177].

### 4.7 Collagen type IX

Collagen type IX is mainly found in cartilage, where it is involved in the maintenance of cohesion between fibrillary and extrafibrillar compartments. In bone, loss of collagen type IX yields disorganization of the trabecular network, which shows increased fibronectin deposition [65]. At the same time, the loss of collagen type IX impairs myeloid differentiation (leading to reduced numbers of myeloid cells) and myeloid cell function including macrophage activation which finally yields a strongly reduced ability of macrophages to clear bacterial infections [65]. Thus, collagen type IX appears to play a role in the hematopoietic compartment, especially in the innate immune system.

### 4.8 Collagen type X

Collagen type X is an element of cartilage, where it is usually detected in the growth plate and the calcified zone of articular cartilage. It is expressed by hypertrophic chondrocytes [230]. Studies with collagen type X deficient mice showed that this collagen type also plays a role in bone microstructure and mineralization as well as in lymphopoiesis. Loss of collagen type X in mice leads to alterations in the trabecular bone matrix. Simultaneously, the inherent trabecular bone cells change their cytokine expression repertoire and thereby lose their ability to support lymphopoiesis [66], [67], [231]. Thereby, collagen type X provides an interesting link between the endochondral ossification during development and hematopoiesis [231].

### 4.9 Collagen type XIV

Expression of collagen type XIV is often found in tissues that express collagen type I at the same time. Direct interactions of collagen XIV were described with perlecan, decorin and collagen type VI, but not with the fibrillar collagen types I, III or V [68], [232], [233]. In human BM, collagen type XIV is expressed by BM stromal cells and appears heterogeneously distributed: some regions are rich and others are free of collagen type XIV. It was demonstrated that collagen type XIV interacts with myeloid and B-lymphoid hematopoietic progenitors, however, mature B-cell lineages were not able to adhere to this collagen. Via blocking experiments the receptors responsible for this interaction could be narrowed down to the class of proteoglycans [68].

### 4.10 Collagen type XVIII

Collagen type XVIII is a member of the group of multiplexins [234]. Collagen type XVIII can be found in many tissues and is an important component of basement membranes of epithelia and endothelia [235]. Degradation of collagen type XVIII by cathepsin K or elastase releases a 22 kDa fragment from one of the non-collagenous domains. This fragment, called endostatin, has diverse biological functions. It can interact with multiple adhesion receptors and vascular endothelial growth factor receptors (VEGFR), and can influence the Wnt signaling pathway. Furthermore, endostatin is able to bind to heparin sulfate chains of proteoglycans via its heparin binding site as well as to nucleolin, tropomyosin or caveolin-1 [234]. Functionally, endostatin inhibits endothelial cells. It is a central player in the regulation of endothelial cell formation, survival, adhesion, migration, proliferation and apoptosis. Thereby, it acts as an angiostatic agent under physiological and pathological conditions, including tumorigenesis. Thus, endostatin is a potential anticancer drug [234].

In BM, collagen type XVIII is mainly detected around arteries, according to a spatial single cell transcriptomic data set of the HSC niche that was recently published [107] together with the referring online tool in which the gene Col18a1 can be searched [236]. Previously it was found that endostatin treatment leads to reduced BM vascularization in mice [214]. In *in vitro* experiments, the matricryptin endostatin was shown to inhibit osteoblast proliferation and matrix mineralization [213], which might lead to changes in osteoblasts’ number which in turn was shown to play a role in the endosteal HSC niche [3], [4]. Furthermore, endostatin inhibits the VEGF-A induced osteoclastic bone resorption and osteoclastic differentiation of HSCs [69] and has thus not only an indirect but also a direct role in the regulation of hematopoietic processes. Accordingly, endostatin has also effects in hematological diseases, particularly acute myeloid leukemia (AML). It has been associated with increased vascularity in the BM of AML patients [237] and elevated serum levels of endostatin with a favorable outcome [238]. Endostatin treatment in AML acts in most cases not directly on the blasts but indirectly via their microenvironment [239] or by inhibition of neo-angiogenesis in AML BM [240].

## 5 Proteoglycans and hyaluronic acid in hematopoietic niches

### 5.1 General

**Fig. 3:**
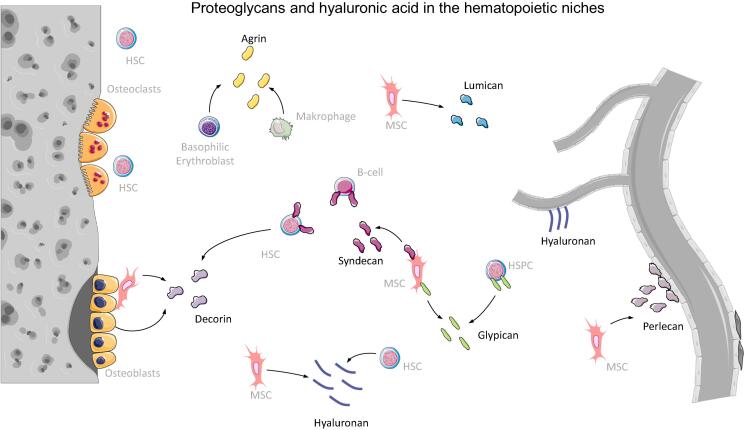
Proteoglycans and hyaluronic acid in the HSC niches. Most abundant proteoglycans of the HSC niche are shown and cells which are able to express the different proteoglycans and hyaluronic acid (hyaluronan). The endosteal niche is shown on the left and the vascular niche on the right. Figure was created with the help of Servier Medical Art by Servier, licensed under a Creative Commons Attribution 3.0 Unported License.

Proteoglycans are the third major ECM component influencing the behavior of HSCs in their niches. Proteoglycans consist of core proteins and glycosaminoglycan (GAG) side chains. These side chains are composed of repeating disaccharide units. Each disaccharide unit consists of a hexosamine [D-glucosamine (GlcN) or D-galactosamine (GalN)] and a hexuronic acid [D-glucuronic acid (GlcA) or L-iduronic acid (IdoA)] or galactose units [241]. The heavily glycosylated proteoglycan molecules can be classified by their size and their GAG side chain composition. The different classes of GAG side chains, which can be attached to a core protein, are keratan sulfate (KS), chondroitin sulfate (CS), dermatan sulfate (DS) or heparan sulfate (HS) [242]. In particular, the family of heparan-sulfated proteoglycans (HSPGs) seems to play an important role in the HSC niche [68], [242], [243], [244]. HSPGs can be found as membrane-bound proteoglycans or as secreted molecules within the ECM. Both forms of HSPGs are able to influence HSC behavior [245]. Besides binding to different core proteins, the structural diversity of HSPGs also arises from sulfate groups attached to different positions of the repeating disaccharide units [241]. In the following part, we will first describe the most abundant proteoglycans of the HSC niches (Fig. 3) before taking a look at putative binding partners of GAGs and how they can serve as potential therapeutic targets.

### 5.2 Perlecan

Perlecan, originally named heparan sulfate proteoglycan-2, consists of a very large core protein of approximately 460 kDa to which three HS side-chains are attached [246]. In the HSPC niches, perlecan is synthesized by human and murine BM stromal cells and deposited in the ECM network [245], [247], [248]. In addition, it is also expressed by K562 cells, a cell line derived from a patient with chronic myelogenous leukemia [248]. Loss of perlecan leads to several skeletal developmental defects [249], but so far a direct effect on hematopoiesis was not reported. Interestingly, perlecan shows an anti-adhesive activity for various hematopoietic cell lines and BM mononuclear cells, but at the same time it presents adhesive properties for fibroblasts and endothelial cells [70]. Perlecan also binds to granulocyte macrophage colony stimulating factor (GM-CSF) and presents it to HSPCs [70]. Binding sites for heparin, nidogen and fibulin-2 have also been observed [250]. Whereas the adhesive interactions of perlecan are mediated through β1-integrins, the responsible factors for the anti-adhesive effects have so far not been identified. Additionally, perlecan has an activating effect on sonic hedgehog signaling [251] and has been proposed to form a network together with collagen type X, which can sequester hematopoietic cytokines, leading to a compartmentalization of the BM microenvironment [242]. Besides GM-CSF binding, HS chains of perlecan are involved in binding of FGF-2 which is crucial for its retention in collagen I scaffolds [252].

### 5.3 Agrin

Another prominent proteoglycan of the HSPG family is agrin. First investigations showed that it is expressed and secreted by neurons and plays an important role in the neuromuscular junction [253]. In the neuromuscular system, Lrp4 expressed by skeletal muscle cells mediates muscle-specific receptor tyrosine kinase (MuSK) activation after agrin binding [254]. In the hematopoietic system Mazzon *et al*. were able to demonstrate that agrin is crucial for HSC – stromal cell crosstalk in the murine HSC niche [71]. In contrast to the neuromuscular system, it was shown that agrin signaling in the hematopoietic compartment is mediated via the dystroglycan receptor and that agrin signaling is required for survival and differentiation of HSC [71], [73]. Furthermore, agrin expressed by erythroid cells and macrophages leads to activation of the receptor tyrosine kinase EphB1 resulting in an upregulation of integrin α5β1 [72]. This may present a mechanism to control cell–cell adhesion and red blood cell development.

### 5.4 Serglycin

Serglycin, also known as “hematopoietic proteoglycan core protein”, is expressed in most immune cells, mainly present in intracellular secretory compartments [255]. Its expression is upregulated during the early stages of myeloblast differentiation and decreased as the myeloid cells mature [256], [257]. Secretion of serglycin is highly regulated in mast cells and platelets, where it can be found in storage granules or secretory vesicles, respectively [258]. In contrast, a constitutively high expression of serglycin can be observed in multiple myeloma cells where it was shown to mediate cell adhesion to BM components [259], [260]. Serglycin was also reported as a marker for AML, distinguishing these cells from Philadelphia chromosome-negative chronic myeloproliferative disorders [261]. Serglycin is associated with megakaryotic differentiation [262], [263] and myeloblast differentiation [256]. In contrast, a down regulation of serglycin was observed during promyelocyte differentiation into mature neutrophils [257]. Serglycin can interact with different matrix molecules, including collagen type IV and fibronectin [264], and CD44 [265], [266].

### 5.5 Small leucine-rich proteoglycans

A very large subfamily of proteoglycans are small leucine rich proteoglycans (SLRPs). All core proteins of this subfamily possess leucine rich repeat domains [267]. A plethora of studies exists investigating the SLRP decorin. The name decorin is derived from the observation that this SLRP “decorates” fibrillar collagen and modulates collagen fibrillogenesis [268]. Several binding partners of decorin and associated functions have been identified [reviewed in [267]]. Ichii and coworkers identified decorin as a regulator molecule in hematopoiesis. In co-culture models with human and murine HSPCs and stromal cells, Wnt3a strongly induced decorin expression in stromal cells that maintained some HSPC characteristics, indicating a functional role of decorin in the HSC niche [75]. Another study investigated the role of decorin in multiple myeloma BM microenvironment and observed that myeloma cells decrease decorin secretion of osteoblasts and propose an indirect antagonistic action of decorin on myeloma cells [269].

The SLRP biglycan is quite similar to decorin showing more than 65% homology [267]. Like decorin, biglycan binds TGF β and modulates TGF β bioactivity *in vitro* [270]. In contrast to decorin, biglycan shows proinflammatory properties by binding to Toll-like receptors (TLR)-2 and -4 [271]. The role of biglycan in the hematopoietic system is still unresolved. HSCs are able to express biglycan, and this expression pattern has been proposed to influence the fate of HSC via TGF β [272]. A transient inhibition of TGF β significantly increases HSCs’ ability to engraft into murine BM [273]. However, depletion of biglycan showed no effect on murine hematopoiesis and HSC function [274].

The SLRPs lumican and fibromodulin carry KS and polylactosamine, an unsulfated variant of KS [267]. Both SLRPs are able to bind to the same region of collagen I, with fibromodulin binding with higher affinity [275]. Lumican is mainly expressed in mesenchymal tissue and tumor stroma and numerous publications analyzed the involvement of lumican in tumorigenesis and inflammation [reviewed in [267], [276]]. As for decorin and biglycan, the impact of lumican and fibromodulin in the HSC niche is not very well known. A few studies investigated the role of SLRPs in hematopoietic disorders. It was shown that CD34^+^ Nalm-6 cells promote chemoresistance by down-regulating lumican expression in MSCs [277]. Interestingly, fibromodulin appears to be selectively expressed in B-cell chronic lymphocytic leukemia and mantle cell lymphoma within the hematopoietic system [278].

### 5.6 Syndecan

Syndecans are single-pass transmembrane proteoglycans, carry HS chains and belong to the group of HSPGs [279]. They can act as receptors and co-receptors, influencing different signaling pathways [280]. Four different isoforms of syndecans have been identified in mammals named syndecan 1–4 [267]. Syndecan-4 is the only isoform that exists not only as transmembrane molecule but also occurs as a soluble isoform following an alternative splicing event [281]. The extracellular domain of all syndecans can be cleaved by sheddases, releasing syndecans into the extracellular milieu. This shedding process can influence other cells in a paracrine manner [282]. Within the HSC niche, syndecan-3 and syndecan-4 are expressed on the surface of marrow stromal cells. It has been suggested that these HSPGs, together with perlecan, are important components building the HSC niche [245]. Recent studies have highlighted that syndecans are not only expressed on BM stromal cells in the HSC niche, but also directly on murine HSPCs and that syndecan-2 is enriched on the surface of long-term murine HSCs [283], [284]. In addition, it has been shown that B-lymphoid and myeloma plasma cells expressing syndecan-1 bind to collagen type I in the malignant HSC niche [57], [225].

### 5.7 Glypican

Glypicans also belong to the HSPG family. Glypicans are bound to the cell surface via a glycosylphosphatidylinositol (GPI) anchor [285]. In mammals, 6 different glypicans are known [286]. Similar to syndecans, a shedding mechanism is known for glypicans. The lipase notum cleaves GPI anchored glypicans and releases proteoglycans into the ECM [287], [288]. To date, the role of glypicans within the HSC niche is poorly characterized. Siebertz *et al*. demonstrated that glypican-4 is expressed on human and murine BM stromal and HPCs [289]. Mice deficient in glypican-3 showed an altered myelopoiesis and impaired osteoclast differentiation [79]. In another study, the inhibitory function of glypican-3 on the dipeptidyl peptidase IV (CD26) was analyzed [78]. SDF-1 can be degraded by CD26. Inhibition of CD26 through glypican-3 leads to an increase of SDF-1, resulting in a higher chemotactic activity of HSPCs as well as enhanced homing and engraftment potential [78]. These findings demonstrate the ability of membrane-bound proteoglycans to strongly regulate and influence the fate of HSPCs.

### 5.8 Binding partners of GAGs

HS chains can bind a large variety of proteins, among them are several growth factors and cytokines which are involved in regulating maintenance, proliferation and differentiation of HSCs [e.g. fibroblast growth factor (FGF), platelet factor 4 (PF4), transforming growth factor β (TGF β) or interleukin-8 (IL-8)] (reviewed in [290]).

FGF plays an important role in the development of the hematopoietic niche in zebra fish [291], and the activity of FGF signaling can be controlled through HS chains. HS is essential for the association of FGF and its receptor [292], [293]. It was also proposed that HS binds GM-CSF and IL-3 and that the HS-bound form of these molecules represents the biologically active form, which is presented to hematopoietic cells in order to regulate hematopoiesis [70], [294], [295].

Furthermore, long-term *in vitro* cultivation of HSPCs can be stimulated through the addition of different proteoglycans. Gupta *et al*. showed that for long term cultivation of HSPCs heparin-sulfated IL-3 and heparin-sulfated macrophage inflammatory protein-1α or PF4 is favorable [296].

HS and CS/DS-GAGs, expressed on the surface of BM endothelial cells, are able to bind SDF-1 in a sulfate-dependent manner. In this way the CXCR4 binding side of SDF-1 is presented to HSPCs [297]. This binding mechanism promotes adhesion and arrest of HPSCs under flow conditions [297]. In the HSC niche, GAGs not only influence HSCs by binding important growth factors or cytokines. Moreover they are involved in the regulation of signal transduction pathways like the Wnt- [298] and Hedgehog-pathways [299], which impact on HSC development and hematopoiesis [300], [301].

### 5.9 Hyaluronic acid (Hyaluronan)

Hyaluronic acid (HA) is a non-sulfated linear GAG of the ECM, which is not bound to a core protein. It consists of disaccharide units of glucuronic acid and N-acetylglucosamine [302]. HA can bind to several receptors as well as to different growth factors and components of the HSC niche [303]. Two of the most important receptors of HA in the HSC niche are CD44 and receptor for hyaluronan mediated motility (RHAMM). CD44 is important for adhesion and maintenance of HSPCs as well as for HSPC migration [304], [305]. RHAMM seems to modulate motility of HSPCs [80]. In general, HA is involved in HSPC mobilization [80] and proliferation [82], [84]. Low molecular weight HA decrease HSPC migration towards SDF-1 [81]. In the HSC microenvironment different cell types are able to produce HA (reviewed in [303]). Murine and human HSCs also express HA, and HA expression affects the distribution of HSPCs after transplantation [82], [83]. HA expression is strongly increased in MSCs from patients with multiple myeloma [306]. Deficiency in HA synthase gene expression leads to a significantly impaired supportive function of MSCs on hematopoiesis [83]. This shows that too little or no HA is detrimental to hematopoiesis, while also increased HA concentrations can have adverse effects such as hematological anomalies [307].

### 5.10 GAGs as potential therapeutic targets in the HSC niche

Several growth promoting and differentiating factors can bind to GAGs which influences the bioavailability of these growth factors for cells of the HSC niche. Thus, GAGs provide a matrix-bound or cell surface-bound reservoir of growth factors by stabilizing them [308], [309]. Due to this feature, GAGs have a great potential as therapeutics. Synthetic GAG mimetics have already been used to regulate the biological activities of growth factors during rat osteogenesis. It was successfully shown that GAG mimetics can influence the proliferation, migration and osteogenic phenotype of rat MSCs *in vitro* [310].

Syndecans can serve as biomarkers and are potential pharmacological targets for the treatment of cancer [282]. Syndecan-1 is well characterized as a marker for multiple myeloma [311]. Its expression is needed for robust growth, vascularization and metastasis of myeloma tumors [312]. Additionally, it was shown that a short peptide, derived from tenascin-C, activates β1 integrins via syndecan-4. These interactions result in apoptosis of diverse hematopoietic tumor cell lines [313]. It is tempting to speculate that syndecan-4 may be a promising pharmacological target.

HA abnormalities have been reported in different hematological malignancies [303]. Multiple myeloma cells showed a HA coating around cells due to different HA synthases expression. B-cells from healthy donors do not show this pericellular HA coating [314]. In addition, it is known that HA contributes to multidrug resistance and that perturbation of HA–tumor cell interactions leads to reduced tumor growth *in vivo* [315]. Several studies reported an increased HA concentration in BM biopsies from AML patients [316], [317]. In this context, the size of HA appears to play an important role. Onoda *et al*. showed that low molecular weight HA (LMW HA), but not high molecular weight HA (HMW HA) reduces drug induced apoptosis in leukemic cells. The authors provide evidence that LMW HA binds to CD44 resulting in a rapid increase in tyrosine phosphorylation of intracellular proteins [318]. The interaction of HA with CD44 on AML cells can induce both cell differentiation [319] or apoptosis [320]. Jin *et al*. transplanted human AML cells in mice and observed that a CD44 specific antibody eradicated AML leukemic stem cells *in vivo* [321]. Another study demonstrated that the disruption of HA–receptor interactions sensitizes primary effusion lymphoma cells to chemotherapy [322]. Lastly, it has been demonstrated that a retinoic butyric HA ester induces apoptosis in retinoic acid resistant leukemic cell lines [323].

Considering all these studies, HA-interactions provide a promising target for the treatment of hematological malignancies and a detailed understanding of HSC-HA interactions is critical for the development of new treatment regimes.

## 6 How HSCs respond to ECM signals via cellular receptors

### 6.1 General

Molecules which mediate cell–matrix or cell–cell interactions are best known as cell adhesion molecules (CAMs). Besides regulating cell adhesion, CAMs can also be involved in signal transduction processes or can act as mechanosensors of the surrounding microenvironment [324], [325]. Six main families of CAMs can be distinguished: the cadherin family, three selectins, members of the immunoglobulin superfamily, the mucin-like family, CD44 and variants, and the integrin family. Most of the CAM families are exclusively involved in mediating cell–cell interactions. The primary cellular receptors mediating signals from the ECM are transmembrane receptors belonging to the integrin family [326]. Other matrix receptors such as dystroglycan [327], BCAM/Lutheran [328], DDR [329] or LAIR [330] are not classified as members of one of the six main CAM families. In the following, the major ECM receptors present on HSPCs will be discussed.

### 6.2 Integrins

The majority of integrins are responsible for cell–matrix interactions, but a smaller fraction of this family is also involved in cell–cell communication [331]. All integrins consist of an α- and a β-subunit which are non-covalently linked to each other on the cell surface. In mammals, 18 distinct α- and 8 β-subunits can form 24 different integrins which can be divided into subfamilies according to their β chains [326]. The largest subfamily is the β1-integrin family with twelve members. The β2 integrin family with four members are called the “leukocyte integrins” since they are almost exclusively expressed on hematopoietic cells [332]. All integrin chains possess a single transmembrane domain and only a short cytoplasmic domain except the β4 chain. The integrins can connect the ECM with the actin cytoskeleton and mediate bi-directional signaling [333], [334]. In this process a plethora of intracellular adaptor molecules is involved [335]. The “outside-in signaling” comprises phosphorylation events and activation of small G-proteins within the cell [336]. On the other side, in “inside-out signaling” intracellular signals can act on the cytoplasmic tails of integrins, resulting in conformational changes of the extracellular ligand binding domain and an altered affinity for ligands [337].

Integrins can be subdivided into smaller subfamilies not only according to their β-subunit, but also according to their ligand specificities [326]. The integrins α3β1, α6β1, α7β1 and α6β4 are receptors for different laminin isoforms. The integrins α1β1, α2β1, α10β1 and α11β1 form the subfamily of integrin collagen receptors. Another key subfamily are the RGD-dependent integrins which comprises the integrins α5β1, α8β1 and the αv-containing integrins. These integrins specifically recognize the short three amino acid motif RGD (Arg-Gly-Asp) found in several ECM molecules, especially in osteopontin or fibronectin [561].

HSPCs are able to express a variety of integrins, and numerous studies have demonstrated an important role for integrins in hematopoietic development [338]. Recently, Tomellini and coworkers found that integrin α3 expression can be used as a late marker for human long-term cultured HSCs [339]. They cultured human cord blood derived CD34^+^ cells in the presence of the pyrimidoindole derivative UM171 and showed that the integrin α3 positive cells exhibit a durable multilineage differentiation ability and integrin α3 is important for long-term engraftment [339]. In contrast, on freshly isolated human BM CD34^+^ cells Gu and colleagues could not detect integrin α3β1 [16]. Prominent expression of the integrin subunits α4, α5 and α6 were detected early on in human HSPCs [16], [340], [341]. It is well documented that both integrins α4β1 and α5β1 are involved in adhesion of HSCs to fibronectin [342], [343], [344], [345]. Furthermore, binding of α4β1 to fibronectin is important in self renewal and survival of HSCs [166], [346]. It was also shown that α4β1 plays a role in preventing early CD34^+^ HSPC apoptosis [347]. Interestingly, this is in contrast to various types of hematopoietic tumor cells where a sustained adhesion to fibronectin via α4β1 induces apoptosis [313]*.* The integrin α4β1 does not only mediate binding of HSPCs to fibronectin, but also to the vascular cell adhesion molecule (VCAM)-1 expressed on endothelial cells. This α4β1–VCAM-1 interaction is important in initial stages of HSC homing to the BM [348]. Hereby, activated α4 integrins expressed by HSCs mediate the strong adhesion to VCAM-1 expressed by BM endothelial cells as a prerequisite for transmigration [349]. The chemokine SDF-1 was shown to be responsible for integrin α4β1 and α5β1 activation on human immature CD34^+^/CXCR4^+^ cells [350]. Furthermore, employing antibodies against integrin α4β1, it could be demonstrated that homing of HSPCs was significantly reduced and that the development of erythroid cells was also inhibited [348], [351].

Furthermore, α4β7 is crucial for integrin-mediated homing following BM transplantation. Here cell–cell interactions mediated by α4β7 and its counter receptor mucosal addressing cell adhesion molecule-1 are responsible for the integrin-mediated homing [352]. These findings were supported by Qian *et al.* who proposed that both the α4 and the α6 integrin subunits are involved in HSC homing [15]. Arroyo and coworkers could show that precursors for B- and T-cells require α4 expression for normal development and that mice deficient in α4 integrins showed an abnormal hematopoiesis [353], [354]. Furthermore, in a conditional-knockout mouse model, HSPCs deficient in the α4 subunit accumulate in the peripheral blood and showed an impaired reconstitution and self-renewal capacity in competitive serial transplantations [355]. Altogether, integrins containing an α4 chain seem to play an important role both in human and murine HSCs in their specific microenvironment.

Integrin α6 subunit can assemble with the integrin β1 or β4 chain, and both α6β1 and α6β4 are receptors for laminin isoforms containing the laminin α5 chain [356]. Integrin α6β1 is prominently expressed both on human and murine HSCs and mediates strong adhesion to LM-511/521 [14], [16]. In contrast, the expression and function of the integrin α6β4 on human HSCs is still unresolved [14]. The α6 subunit, also termed CD49f, was identified as a specific marker for human HSCs, and single CD49f^+^ cells were shown to be very efficient in long-term multilineage engraftment [357]. However, integrin α6 is not only a marker for human HSCs, it is also expressed in many other adult multipotent stem cell types leading to the suggestion that integrin α6 is a reliable and authentic general stem cell marker [358]. Of note, the laminin ligands of integrin α6 are also found in many somatic stem cell niches, including colonic [359], corneal [360], epithelial [361], hematopoietic [16], [143] hepatic [362], hair follicle [363], neuronal [364], [365] or spermatogonial [366] niches, indicating an important involvement of the LM-511/integrin α6–axis in adhesion and self-renewal of multipotent stem cells in their appropriate microenvironment.

In addition, transcription of integrin subunits α7-α11 have been systematically analyzed in human CD34^+^ HSPC [189]. This expression-screening revealed that integrin α9β1 is strongly expressed on human HSPCs and contributes to the adhesion to osteoblasts as well as differentiation of HSPCs in the endosteal niche [189]. In agreement with these results are findings from the group of Susan Nilsson who showed that murine as well as human HSCs express not only integrin α4β1, but also α9β1 and that trOPN can bind to both integrins. In the BM niche, binding of trOPN to these integrins regulates attraction, retention and the release of HSPC [35]. Recently, a strong expression of the integrin α7 on human CD34^+^ HSPC could also be detected [147], however a functional involvement of this receptor in adhesive interactions has still to be resolved.

Integrin αIIbβ3 is mainly expressed on platelets, where it can be rapidly activated. In its activated state it serves as a receptor for ligands that can bridge platelets together [367], [368]. This important function of αIIbβ3 predestines it as a therapeutic target. Interestingly, in mice expressing a talin-1 mutant, talin-1 binds αIIbβ3 without activating it, resulting in an impaired hemostasis [369]. Moreover, αv integrins can also act as modulators of effects induced by the surrounding microenvironment. The murine integrin αvβ3 intensifies IFNγ-dependent responses of HSCs [370], [371], and the αv subunit can also regulate HSC proliferation via interaction with periostin [42].

Several integrin β-subunits have also been studied in HSPCs in more detail. The β1 integrin chain of fetal and adult HSCs is involved in colonizing the fetal liver, spleen, and BM [372]. It was shown that the absence of β1 integrin on HSCs impairs the migration but not the differentiation into different lineages [373]. Brakebusch and coworkers showed that in the murine system the integrin β1 chain is not essential for normal hematopoiesis but that it plays a pivotal role in the T-cell dependent IgM antibody response [374]. Furthermore, in an RNAi study with primary human cord blood-derived HSPCs, the guanine nucleotide exchange factor cytohesin 1 was identified as an important regulator of β1 integrin-dependent adhesion and engraftment [375]. β2 integrins have also been studied in the HSC microenvironment in greater detail. β2 integrins alone do not seem to be absolutely essential for homing of HSCs but a synergistic effect with integrin α4β1 was observed [376]. Interestingly, murine HSPCs do not express the integrin αLβ2 [377].

The β3 integrin subunit was reported to correlate with properties of quiescent HSCs, especially when the β3 chain was linked to the αv chain [378]. The β7-null mice showed no obvious defects in lymphocyte development [379], while human cord blood progenitors showed an induced expression of the β7 subunit accompanied by a downregulation of β1 and α5 integrins during eosinophilic differentiation [380].

The interaction of integrins and components of the ECM can lead to the formation of focal complexes and can further maturate into focal adhesions (FAs) which anchor the actin cytoskeleton network to the ECM [381]. So far, mature FA structures have not been observed for HSCs although HSCs are able to express the FA proteins zyxin [382] and kindlin-3 [383], [384]. Kindlin-3 activates integrins through binding to β integrin tails resulting in an enhanced ligand affinity [385]. A deletion of kindlin-3 in mice results in leukocyte adhesion defects and osteopetrosis [386], [387]. Similarly, in humans, a loss of kindlin-3 leads to leukocyte adhesion deficiency type III [388], [389]. In mice, retention of activated and proliferating HSCs in the niche depends on the expression of kindlin-3. Instead, kindlin-3 seems to be dispensable for quiescent HSC [383]. Talin connects β integrin subunits to the actin cytoskeleton. Silencing of talin-1 revealed that adhesion of HSC is talin-dependent [390]. Further downstream, the FA kinase (FAK) plays an important role in signal transduction. In HSCs, the FAK homologue Pyk2 has also been identified to be involved in signal transduction processes [391].

Integrins are well-studied as therapeutic targets [392], [393], [394], [395]. Several drugs targeting integrins including monoclonal antibodies, peptides or small molecules are already on the market and more clinical trials are on the way [395]. Therapeutics targeting the lymphocyte integrins α4β1 and α4β7 are indicated in multiple sclerosis and inflammatory bowel disease [396]. The integrins α4β1/α9β1 can be targeted with a small molecule called BOP (*N*-(benzenesulfonyl)-L-prolyl-L-*O*-(1-pyrrolidinylcarbonyl) tyrosine) leading to a rapid mobilization of long-term multilineage reconstituting HSCs [397]. The following antagonists against αIIbβ3 are used as therapeutics (here their commercial names are given): Abciximab™, Eptifibatide™, and Tirofiban™. They are mainly used for the prevention of periprocedural thrombosis in percutaneous coronary interventions [367]. It is more than likely that further research will identify additional applications for targeting integrins to treat different malignancies of the hematopoietic system.

### 6.3 Other non-integrin ECM receptors on HSCs

A major non-integrin receptor on HSPCs which can interact with various ECM components is CD44 [398]. Due to insertion of alternatively spliced variable exon products and different post translational modifications CD44 exists in several variant isoforms (CD44v) [399]. The standard form CD44s is the smallest isoform. In the adult organism CD44s is almost ubiquitously expressed, but the highest expression is found on hematopoietic cells, especially on HSCs [400]. The variant CD44v6 is also expressed on HSPCs, but at a low level [401]. Another variant, CD44v7, is found on BM stromal cells and can support HSPC homing [401].

CD44 is the major HA receptor, but it can also bind to osteopontin, fibronectin, collagen types I and IV and the laminin isoform LM-111 [400]. The adhesive interactions are mediated by the N-terminal globular domain which is found in all CD44 variants. Upon CD44 binding and activation by HA, integrin expression on HSPCs can be up-regulated thereby strengthening adhesive interactions in the niche [402]. The HA-mediated integrin α4β1 up-regulation promoted stronger adhesion to both fibronectin and the membrane-bound ICAM-1 [403]. The isoforms CD44v6 and CD44v10 contain specific binding sites for osteopontin [404]. Thus through CD44-mediated interactions between HSPCs and the BM microenvironment, CD44 can directly contribute to the regulation of HSC homing, engraftment, quiescence and prevention of apoptosis, and it can also be involved in the development of hematological neoplasms.

Dystroglycan (DG) and BCAM/Lutheran are non-integrin laminin receptors mainly interacting with laminin isoforms containing the α5 chain [328], [405]. DG consists of two subunits, a membrane-spanning β -DG subunit and a highly glycosylated extracellular α-DG subunit [405]. A prominent expression of the α-DG subunit could be detected on human CD34^+^ HSPCs [406], however our knowledge about a functional involvement of DG on HSCs in their niches is still fragmentary. BCAM/Lutheran, also known as CD239, are two isoforms of the same gene only differing in their cytoplasmic domains [328]. So far, BCAM/Lutheran expression was only found on late erythropoietic progenitors [407], but newer results now show that BCAM/Lutheran is already expressed on human CD34^+^ HSPCs and plays a role in migration and differentiation of CD34^+^ HSPCs [147].

LAIR-1, also known as CD305, belongs to the leukocyte receptor complex [330]. In its cytoplasmic domain it contains two immunoreceptor tyrosine-based inhibitory motifs (ITIMs) probably regulating the tyrosine phosphatase SHP-1. LAIR-1 is a non-integrin collagen receptor strongly expressed on human CD34^+^ HSPC, but whether this adhesion receptor is functionally involved in regulating HSCs in their niches is still unknown [408]. Another receptor type for collagens are DDR-1 and DDR-2. On mature leukocytes a prominent expression pattern of DDR-1 could be detected, but when the expression of DDR-1 starts during leukocyte development is not yet known [409]. DDR-2 has been reported to be expressed on BM MSC contributing to bone formation and BM adipogenesis [410], but whether it plays a role in the hematopoietic niches is also still unknown.

## 7 Changes in the ECM of hematopoietic niches during development

Different niches harboring HSCs at varying developmental stages have specific characteristics that trigger the correct development of HSCs. Besides different intrinsic expression patterns of HSCs during development, the microenvironments differ and contribute to the needs of HSCs. A highly orchestrated interplay of different cells and ECM molecules in the HSC niches is crucial. When analyzing the varying microenvironments during HSC development, most studies focused on the different composition of cells and soluble factors [411], [412]. Cell–cell as well as cell–matrix interactions have been investigated during HSC developmental trafficking. For the migration of HSCs to the fetal liver, VE-Cadherin, αIIb integrin, β1 integrin, c-KIT and CXCR4 are vital [413]. Adhesiveness of HSPCs to ECM molecules during the different developmental stages — fetal liver, umbilical cord blood, adult BM — has been analyzed [414]. In comparison to HSPCs from adult BM, HSPCs from umbilical cord blood showed a higher adhesion to fibronectin, whereas HSPCs derived from fetal liver showed an impaired binding [414]. Similar to adult BM HSPCs, the adhesion of fetal liver HSPCs to fibronectin is mediated by α4β1 and α5β1 integrins. However, expression of α5 integrin is higher in fetal liver HSPCs compared to BM HSPCs suggesting that this integrin may be in a low affinity state in the fetal liver cells. The expression of α2 integrin seems to be only important in fetal liver-derived HSPCs, and it was suggested that integrin α2β1-dependent adhesion to collagen type IV is crucial for developmental stage-specific regulation in fetal liver HSPCs [414]. For the colonization of fetal liver, spleen or bone marrow expression of β1 integrin is absolutely necessary [372]. Furthermore, it was demonstrated that integrin α4 is important during homing of fetal liver HSPCs in mice [15]. Consequently it was shown that the small molecule inhibitor firategrast which inhibits the integrins α4β1 and α4β7 had a mobilization effect on HSCs from the fetal liver [415]. The authors also observed an increased *in utero* allogeneic engraftment of HSCs mobilized from fetal liver in the murine system. Another study investigating adhesion molecules of fetal liver HSCs provided evidence that human fetal liver HSCs express GPI-80 and integrin αM and that both surface molecules are important for *in vitro* expansion and engraftment [416]. Such specific characteristics are not restricted to the fetal liver niche. An expression analysis of ECM molecules in the human placenta revealed a specific ECM composition in the first trimester and term tissue that implicates a different impact on HSCs [417].

## 8 ECM remodeling and ECM degrading proteases

Proteolytic enzymes of the metzincin family can degrade and remodel ECM components which can not only affect the structural integrity, but also the biomechanical characteristics of the BM [418]. Metzincins are characterized by a metal ion in their active center and belong to the large metalloproteases superfamily [419]. The metzincins comprise secreted and membrane-bound matrix metalloproteinases (MMPs), membrane-bound ADAMs (a disintegrin and metalloproteinase) and secreted ADAMTS (ADAMs with thrombospondin motifs) with overlapping substrate specificities [420], [421], [422]. The metalloproteinase activities can be regulated by four specific tissue inhibitors of metalloproteinases (TIMPs) [423]. A highly proteolytic microenvironment in the BM can be found during iatrogenic stem cell mobilization [424], [425]. However, these proteases are not only used for matrix remodeling, they can also interfere with cell–cell or cell-matrix interactions by shedding membrane-associated receptors, and they can release and/or process chemokines and cytokines such as the membrane-bound SCF or the secreted CXCL12 thus affecting the bioavailability of the different factors.

### 8.1 MMPs in the bone marrow

Based on their primary structure and on substrate specificities, the MMP family can be subdivided into membrane-type MMPs and the secreted collagenases, gelatinases, stromelysins and matrilysins [418]. The well-studied gelatinases MMP-2 and MMP-9 are not only expressed by almost all microenvironmental niche cells, but also by HSPCs [426]. However, a striking species-specific difference was observed for MSCs. Whereas murine MSCs express MMP-9, this gelatinase was not detectable in human MSCs which only express MMP-2 [427]. Another widely expressed MMP in the BM is the membrane-bound MMP-14 (also designated MT1-MMP) found on HSPCs, MSCs, endothelial cells, stromal fibroblasts and macrophages [426]. On the contrary, the expression of the collagenase MMP-8 seems to be more restricted. MMP-8 is mainly found in neutrophilic granulocytes [428], and to a lesser extent in monocytes and macrophages [429].

MMPs in general are expressed as zymogens (latent precursors) that have to be activated by limited proteolysis with extracellular proteinases such as plasmin or intracellularly with furin. The membrane-anchored MMP-14 is able to activate secreted pro-MMPs thus localizing the degradation of the ECM at the pericellular region [430]. Substrate specificities of the different MMPs can widely overlap, but they also show specific interactions. The gelatinases MMP-2 and MMP-9 can digest different collagen types, they both degrade fibulin-2, but not fibulin-1 [30], however they differ in degrading tenascin-C. The large isoform of tenascin-C, HxB.L, is resistant to MMP-9, but it can be digested by MMP-2 [431]. The collagenase MMP-8 can digest a variety of different collagen types, in addition it can cleave the α5 chain of laminin isoform LM-511, whereas the gelatinases are unable to process LM-511 [428]. The membrane-anchored MMP-14 can process the pericellular matrix by degrading different collagen types, by activating pro-MMPs and by acting as a sheddase digesting membrane-bound receptors such as syndecan-1 or CD44 [432]. Furthermore, hematopoietic niche cell-MMP-14 can control postnatal blood formation by activating hypoxia-inducible factor-dependent niche factors essential for terminal differentiation of mature blood cells [433].

Another important function of MMP-2, -8, -9 and -14 in the BM is their ability to digest critical growth factors. All four MMPs can inactivate CXCL-12 by removing three or four N-terminal amino acids which are needed for receptor binding [428], [434]. Intact CXCL-12 binds to the receptor CXCR-4 on HSPCs, and this interaction is essential for the homing and engraftment of HSPCs in their niches [435]. The proteolytic breakdown of the CXCL-12/CXCR-4 axis is a prerequisite for the mobilization of HSPCs out of the BM into the peripheral blood. Notably, mobilized HSPCs express a higher amount of MMP-14 on their cell surface in comparison to the quiescent, non-mobilized counterparts in the BM niches [434]. Another growth factor processed by MMPs is the membrane-bound form of SCF, which, as well as its receptor c-KIT (CD117), can be cleaved by MMP-9 which then leads to the mobilization of HSPCs [436], [437].

Compared to MMPs much less is known about the role of the sheddases of the ADAM family in hematopoiesis. Weber and colleagues studied the role of ADAM-10 in a murine deletion model. The ADAM-10^−/−^ mice were characterized by enhanced granulocytic subpopulations and extramedullary erythropoiesis resembling an unclassified myeloproliferative disorder [438]. Clinical markers for myeloproliferative disorder such as elevated TIMP-1 levels in blood plasma were observed in these mice. This study strongly suggest that ADAM-10 is necessary for a balanced myeloid/lymphoid cell-fate decision of HSPCs [438].

### 8.2 TIMPs in the bone marrow

The four mammalian tissue inhibitors of metalloproteinases (TIMP1-4), originally identified as collagenase inhibitors, are secreted proteins that can inhibit all activated MMPs [423], [439]. Whereas TIMP1-3 are widely expressed in hematopoietic and non-hematopoietic cells of the BM, TIMP-4 shows a more restricted expression pattern. The four TIMPs show a roughly 40% sequence identity with each other, with TIMP-2 and TIMP-4 sharing the highest similarities. Among the four family members, TIMP-3 is unique in possessing specific domains that can interact with components of the ECM, a feature not displayed by the other three TIMPs [440]. Therefore, TIMP-3, unlike the other TIMPs, shows a predilection for attachment to matrix components.

Beyond their protease inhibitory functions all TIMPs show protease-independent activities. One of the earliest findings was an erythroid-potentiating activity of TIMP-1 and TIMP-2 [441], [442]. Later it was shown that TIMP-1 can strongly influence hematopoietic cell survival and proliferation [443]. TIMP-1 signaling via the tetraspanin membrane receptor CD63 can stimulate cell survival and proliferation of CD34^+^ HSPCs as well as myeloid progenitor cells [444], [445]. A similar, CD63-mediated effect was observed for AML cells. Here, TIMP-1 promoted cell survival by recruiting the leukemic cells into the cell cycle [446]. On the contrary, proliferation of BM-derived MSCs could be strongly inhibited by TIMP-1, at least partially through the Wnt/β-catenin pathway [447]. TIMP-3, on the other hand, is highly expressed by BM MSCs as well as osteoblasts and this high expression can be used to stimulate the entry into cell cycle of quiescent HSPCs [448], [449]. Whether all of these protease inhibitor-independent activities of the different TIMPs could be harnessed for *ex vivo* expansion of HSPCs or used clinically has still to be studied in more detail.

## 9 Biophysical signals transmitted by the ECM in HSC niches

### 9.1 General: Biophysical parameters influencing HSCs

In the HSC niche, the ECM acts in several ways to control HSC behavior. The mechanisms of action of the ECM in this entity can be categorized as either biochemical — including all signals provided by the chemical nature of the ECM such adhesive sequences or growth factor binding that were described in detail for the individual molecules above — or as biophysical in nature.

Only roughly a decade ago, it became evident that HSCs are sensitive to physical signals in their environment such as shear stress or substrate elasticity [450], [451]. Since then, HSCs were reported to be responsive to many biophysical parameters including nanopatterning (e.g. [452], [453]), nanotopography (e.g. [454]), 3D architecture (e.g. [455]), shear stress (e.g. [450], [456]), hydrostatic pressure (e.g. [457]), mechanical unloading by microgravity (e.g. [458]), and mechanical properties of their surroundings (e.g. [451], [459], [460]). Here, we will focus on the biophysical signals transmitted by the ECM to HSCs in their niche, which are elicited by the structure of the ECM on the nano- and macroscale as well as its mechanical properties.

### 9.2 Mechanical properties of the matrix

As described above for the individual ECM molecules, the expression pattern of them in the BM is heterogeneous. Similarly, the stiffness of the ECM in the BM is also not homogenous [461] and thus differs depending on the particular region or microenvironment observed. The marrow region is very soft with 0.3 kPa [462]. Intermediate values of roughly 0.5–2 kPa and 5–8 kPa were reported for endothelia and vessel walls, respectively [463], [464], [465], and the osteoid matrix formed by osteoblasts at the endosteum is comparatively stiff with 35 kPa [466]. Amongst other ECM molecules and enzymes, collagen type I and III as well as the enzyme lysyl oxidase (LOX) might be partially responsible for the observed stiffness differences throughout the BM. It was shown that the stiffness of the ECM increases with increasing collagen type I concentration, while additional presence of collagen type III in collagen type I structures leads to decreasing stiffness [467], [468]. Additionally, upon secretion, the enzyme LOX catalyzes the oxidative deamination of lysine and hydroxylysine residues in collagen matrices which yields aldehyde groups that can spontaneously crosslink. The crosslinking leads to an increased matrix stiffness [469], [470]. LOX upregulation was observed e.g. in hematological malignancies such as myeloproliferative disorders [471], [472]. Furthermore, it is possible that ageing might lead to alterations in mechanical properties of the ECM in the BM, similarly to observations made in other tissues such as skin [132]. Thus, the stiffness of the ECM in the BM does not only vary locally but also with time as it is influenced by physiological processes such as collagen deposition and enzyme secretion. As these processes can change during certain pathologies [471], [472] and stiffness is known to impact cell behavior [132], it seems possible that ECM stiffness in the BM might also play a role in the development and progression of such diseases.

The first report on the potential influence of matrix elasticity on HSCs was published in 2010 [451]. Holst et al. reported an enrichment of KLS cells when culturing whole BM mononuclear cells on tropoelastin-coated tissue culture plates due to the elastic properties of the used molecule. However, due to the experimental setup the study could not resolve whether the observed effects of substrate mechanics on HSCs are directly elicited by the interaction of HSCs with tropoelastin or if the effects are indirectly transmitted to HSCs via other reportedly mechanosensitive cells in the applied whole BM mononuclear cell population such as MSCs [460].

In the following years, various groups used different types of hydrogels, made from natural macromolecules such as collagen and heparin or synthetic polymers such as poly(ethylene glycol) (PEG), to show the mechanosensitivity of HSPCs. Applying such hydrogels, we could show that human HSPC adhesion and migration are fostered on stiffer fibronectin-coated hydrogels in comparison to softer ones [459]. Fitting to this finding, the Harley group observed stronger cytoskeletal development and spreading on stiffer than on softer collagen gels [460]. Furthermore, they showed that HSPC viability is greater on softer matrices [460]. On gels mimicking the endosteal region of the BM with fibronectin coating and stiffness of 44 kPa rather early myeloid progenitors were maintained while on gels reconciling the vascular niche with laminin coating and lower stiffness of 3 kPa differentiation particularly in the erythroid direction was fostered [56]. Similarly, Chitteti *et al.* reported that murine HSCs are more quiescent on stiffer collagen matrices [473]. 3D culturing of HSPCs by embedding them in starPEG-heparin hydrogels also led to greater quiescence accompanied by lower proliferation and higher stem cell maintenance in the stiffest applied hydrogels [474]. However, when culturing HSPCs on top of these gels in 2D, no effects of stiffness on proliferation or differentiation could be observed. The explanation for the discrepancy of the latter result to the above described 2D studies might lie in the different ranges of stiffness investigated in the different studies. The stiffest hydrogel in the latter study was with 3 kPa in the range of the softer gels in the studies described before. Thus, it appears that the range of stiffness to which HSPCs respond is dependent on the dimensionality of the environment.

In their niches, HSCs are in the direct vicinity of mechanosensitive cells. Since the seminal study by Engler et al. MSCs are known to react to differences in matrix stiffness with altered differentiation [475]. Endothelial cells were shown to stiffen in response to stiffer matrices, which facilitates transmigration of neutrophils through endothelial cell layers [476]. Besides MSCs and endothelial cells, further mechanosensitive cells, including osteoblasts and pericytes, are found in HSC niches. All of these cells closely interact with HSPCs via paracrine signaling that might be changed by mechanical stimulation [477]. It was shown that the cytokine profile secreted by MSCs changes when cells are grown on substrates with different mechanical properties. Via these changed cytokine profiles, priming MSCs on soft polydimethylsiloxane substrates (1 kPa) leads to support of HSPC expansion in co-cultures while priming on stiffer matrices yielded myeloid differentiation of HSCPs [478]. Also culturing MSCs in alginate hydrogels leads to altered cytokine secretion depending on the hydrogel stiffness. The changed cytokine repertoire yielded higher percentages and numbers of Lin^−^ CD45^+^ cells when co-cultured in transwell format with MSCs encapsulated in softer hydrogels (3 kPa) than in stiffer hydrogels (18 kPa) [479].

How do HSCs sense the mechanical properties of the ECM in their environment? The field of mechanosensing in HSCs is still unexplored in large parts. The receptors for transmitting a mechanical signal from the HSCs’ exterior via the membrane to its interior are most likely integrins as they recognize many of the ECM molecules that were used in studies, in which the mechanosensitivity towards stiffness was phenomenologically observed as described above. Besides, other receptors that are known to be mechanosensitive might be involved including adherens junctions proteins, G protein coupled receptors or ion channels [477]. Inside of the cell, myosin motors, cytoskeletal linking, polymerizing and regulating elements, cytoskeletal filaments, caveolae, the transcriptional cofactors YAP/TAZ, the nuclear lamina including lamins A and B and other signaling molecules are potentially parts of the mechanosensing process [132], [477]. Of these candidates, particularly the role of YAP/TAZ, lamins and non muscle myosins was investigated in HSPCs in more detail [462], [480], [481], [482], [483]. Shin et al. showed that non muscle myosin II plays an important role in sensing of the niche and polarized divisions by HSPCs [462]. The same group also found that the ratio of lamin A to B in the nuclear lamina determines the viscoelasticity of this structure, which influences the ability of HSPCs and their progeny to transmigrate through microporous barriers [480]. Also, the ability of mature hematopoietic cells, particularly granulocytes, to migrate through collagen type I barriers, was shown to be impaired by enhanced lamin A expression levels [484]. Nevertheless, it might be that mechanosensing processes change during the differentiation and maturation of cells. This hypothesis is supported by the comparison of two other studies, one of which showed that YAP plays an important role in the sensing of biophysical forces transmitted by blood flow during the formation of embryonic HSCs from hemogenic endothelium [483], while in the other study the authors found that in adult hematopoiesis YAP and TAZ appear to be expendable [485]. All in all, while some progress is made in understanding biophysical regulation in HSCs in general, the question how HSCs specifically sense mechanical properties of the ECM in their niche remains unanswered and is yet to be explored.

### 9.3 Nanostructure of the ECM

The ECM is highly structured not only on the macroscopic but also on the molecular and, thus, the nanometer scale. For cells being in contact with the ECM this means that they sense besides the afore described biochemical composition and mechanical properties of the ECM also its nanostructural features [132]. In comparison to anchorage-dependent cells such as fibroblasts, MSCs or osteoblasts, relatively little studies explored the influence of such features on HSPCs.

A first indication of the sensitivity of HSPCs towards nanostructural features in their environment was provided in 2006, when Chua et al. showed that adhesion and expansion of HSPCs are enhanced on amino-functionalized polyethersulfone nanofibers in comparison to standard tissue culture plastic [454]. Thereafter, nanofibers were often used to mimic the ECM for HSPC expansion as reviewed in [486]. In the following years, it became clear that the lateral distance between adhesive ligands on the nanometer scale influences HSPC adhesion, lipid raft clustering and adhesion receptor distribution in the cells’ membrane [452], [453], [455]. Similarly the density of collagen ligands was shown to influence HSPC viability [460]. Also differentiation and proliferation of HSPCs are affected by nanopatterning of ligands, when in addition to adhesive motifs derived from ECM molecules also ligands of cell–cell-interactions are offered to the cell [487]. However, for cellular ligands, being in contrast to ECM ligands naturally not laterally fixed but movable within the fluid membrane of the signaling cell, it appeared that ligand density rather than nanopatterning are important for regulating HSPC responses [487]. All in all, it seems that more than one signal at a time is needed to instruct HSPC maintenance and differentiation via engineered surfaces, which constitutes a need for novel strategies for bioorthogonal functionalization of biomaterials with several bioactive molecules. Such strategies can be successfully employed to enhance HSPC proliferation, as demonstrated in [488]. Similarly, biomaterial-based approaches for targeted T-cell differentiation from HSPCs without feeder cells necessitate the combination of ECM- and cell-derived signals [489], [490].

While nanofibers have proven to be effective for enhancing HSPC expansion [486], the way in which nanotopography acts on HSPC proliferation and differentiation is not well explored and HSCs might sense such nanotopographical features of culture surfaces either directly or indirectly via adsorbed proteins. Lastly, similar to stiffness-effects, also nanotopography and/or nanostructure might act indirectly via e.g MSCs or osteoblasts in their vicinity, for which the effects of these parameters on cell behavior are well established [491].

### 9.4 3D macroscale structural architecture of the ECM

The HSC niche in nature is a 3D entity. The ECM in this 3D environment acts on HSPCs by embedding them from all sides and thus leading to space constraints, increased matrix availability and higher cell densities than in 2D. Furthermore, the 3D matrix around cells leads to limited diffusion and establishment of gradients of soluble factors, which enables efficient auto- and paracrine signaling between cells, and allows a 3D organization of cells [132], [492]. All of these factors contribute to the effects of the 3D ECM on HSPCs. In order to achieve a more natural behavior of HSPCs, many attempts have been made to create more *in vivo*-like environments for HSPCs. The first step towards this goal is to understand the way in which a 3D environment influences HSPC behavior.

For this purpose, HSPCs were encapsulated in hydrogels of natural or synthetic polymers (examples are [460], [493], [494]), seeded into macroporous (e.g. [495], [496], [497], [498], [499]) or fibrous scaffolds (e.g., [500]), grown in spheroids (e.g. in [501], [502]), cultured in microcavities (e.g. [503], [504], [505], [506], [507]) or subjected to combinations of the different approaches (e.g. [508], [509]). Here, we provide only examples for the different techniques, for a more comprehensive overview concerning this topic the reader is referred to [492], [510].

Nanofibrous scaffolds are widely used for 3D cultures of HSPCs [486], however, as cells are often not able to penetrate deeply into the applied fiber meshes, they should be regarded as pseudo-3D [492]. To overcome this limitation, nanofiber meshes were layered [511] or were combined with macroporous scaffolds [508], [509]. Similarly, microcavities that host HSPCs in culture are not *per se* a 3D environment, as the cells are in a limited volume, but their contact is limited to the bottom and side walls of the well and not taking place in all 3 dimensions. However, when the cells in these cavities are grown to sufficiently high densities to form 3D cell aggregates, they experience a 3D environment within the aggregate. Nevertheless microcavity materials have greatly contributed to our understanding of HSPC regulation by 3D constraints [503], [504], [505], [512]. It was shown that HSPCs cultured in smaller cavities proliferate less and maintain higher levels of stem cell markers than HSPCs grown in larger cavities [512]. In addition, cell encapsulation studies revealed that HSPCs within starPEG-heparin hydrogels proliferate more in a softer and less in a stiffer environment. The authors observed that the cells did not degrade the hydrogel matrix around them but rather compressed it while they were growing [474]. The mechanism in which spatial constraints affect HSPCs might be twofold. First, the cells might physically sense or be physically restrained by the smaller space around them and, second, secreted molecules are more concentrated in smaller volumes [492]. The second effect — the accumulation of secreted molecules in smaller volumes — was also shown to be responsible for the beneficial action of macroporous scaffolds on HSPC expansion in co-culture with MSCs [497]. The HSPCs cultured together with supporting MSCs in the macropores of the scaffolds experience more efficient auto- and paracrine signaling, as the macroporous scaffolds limit the diffusion of secreted molecules, which are thus concentrated within the small volumes of the pores and not diluted out in large volumes of medium as it occurs in conventional 2D cultures [497]. Similarly, it was shown that the effect of the 3D matrices on diffusive biotransport by influencing autocrine feedback signaling of HSCs and paracrine signaling in co-cultures with MSCs or Lin^+^ cells is an important parameter to be considered when developing biomimetic culture approaches for HSPCs [513], [514]. In these studies, the poroelastic properties of the applied hydrogels were correlated to the diffusivity *in vitro*. When considering a potential relevance of these results for *in vivo* applications, the diffusivity of molecules, including not only natural signaling molecules but also drugs in the BM, might be strongly influenced by biophysical properties of the ECM. This hypothesis is supported by the finding that LOX as an important regulator of ECM stiffness via its crosslinking activity is also involved in modifying the physical barrier function of the ECM in 3D for small molecules including drugs [515]. In this way, inhibiting LOX led to improved drug diffusion and efficacy [515].

Lately, 3D printing was applied to create scaffolds for HSPC culture [509], [562]. 3D printing and bioprinting allow to create more complex biomaterials and scaffolds and will be powerful techniques in order to further investigate effects of 3D architecture on HSPCs and its role in drug transport in the BM in the future.

### 9.5 Association of biophysical properties of the ECM in niche with BM pathologies

Dysregulation of the ECM homeostasis in the BM including exaggerated deposition of ECM, enhanced crosslinking activity and deficient ECM remodeling can lead to increased stiffness. These processes materialize during BM fibrosis that goes along with impaired organ function, particularly blood cell production. The association of the observed fiber accumulation with underlying disorders is best investigated for primary myelofibrosis, a myeloproliferative neoplasm [516]. Amongst others, ineffective hematopoiesis in the BM accompanied by extramedullary hematopoiesis and splenomegaly are indicative for this disease. Such enhanced fiber deposition is also found in hematological malignancies such as acute lymphoblastic leukemia, myelodysplastic syndrome and chronic myelogenous leukemia, as well as many other disorders affecting the BM including autoimmune, infectious or inflammatory diseases, exposure to toxins or radiation [472], [516]. Thus, having in mind the substantial effects that matrix stiffness and the fibrous ECM structure from the nano- to the macroscale can have on cell behavior, increased stiffness and changed fiber structure caused by the dysregulated ECM might play a role in disease progression in all of these pathologies. Accordingly, Shin and Mooney found that varying matrix stiffness influences proliferation and sensitivity against chemotherapy of AML cells and is thus a pathologically relevant parameter in such hematological malignancies [517].

## 10 The ECM in artificial stem cell niches

### 10.1 General: Artificial stem cell niches in drug research

During the last two decades an increasing amount of research was dedicated to the development of artificial HSC niches for several applications. In this attempt only limited attention has been paid to research on artificial systems that mimic the HSC niche for drug research despite the facts that (i) the hematopoietic system is a sensitive target for many drugs, (ii) current *in vitro* models are often too oversimplified to reconcile complex responses to drugs and (iii) animal models have limited predictivity in the hematopoietic system due to species-related differences [518]. Engineering artificial stem cell niches requires to develop a system that allows dynamic control of interactions between cells, availability of cytokines and growth factors in matrix-bound or soluble form and the provided ECM [519]. In this endeavor the ECM is often regarded as the part of the niche that is the easiest to be mimicked [520]. In the following, we want to shed light on the questions if this is true and how mimicking the natural ECM evolved from simple coating strategies toward complex systems for investigating potential toxic effects of drugs on the hematopoietic system or evaluating their efficacy in models of diseased BM.

### 10.2 Mimicking the ECM *in vitro*: From 2D to 3D

In the natural niche, the ECM is not only a structural element but also regulates the cell behavior from cell attachment and migration via cell cycling and proliferation all the way to stem cell maintenance and differentiation. The ECM exerts its function via its biochemical and physical properties as elaborated in the chapters above. Therefore, mimicking the ECM in artificial niches requires reconciling biochemical and physical parameters characteristic for the ECM in the niche in order to obtain a fully functional artificial ECM mimic [521].

Many approaches have been used to improve HSC cultures by enabling interaction with ECM *in vitro*. The easiest way is to coat surfaces with ECM molecules, which enhances HSPC culture in comparison to simple suspension cultures without any directed possibility for cell–matrix interaction [522]. Such surface functionalization were conducted using full-length ECM proteins (e.g. fibronectin, collagens or laminins), protein domains (e.g. CS-1 domain of fibronectin) or peptides representing short bioactive motifs of ECM proteins such as RGD or LDV that are minimal integrin recognition motifs [510]. However, not only the pure presence of an ECM ligand but also its spatial presentation in terms of orientation of the ligand or nanopatterning to ensure efficient cell stimulation have to be considered when mimicking the ECM [452], [453], [510]. The next level of complexity is introduced by considering topography of the ECM and transferring cell cultures from a flat 2D system to “2.5D”. Microwell systems and nanofiber substrates are widely applied for this purpose as described above [454], [486], [512], [522], [523], [524].

For mimicking the ECM in 3D, a tumor-derived matrix from Engelbreth-Holm-Swarm mouse sarcoma cells — commercially available under brand names Matrigel or Cultrex — is most widely used. This matrix is rich in basement membrane components, mainly collagen type IV, laminins (mainly LM-111), perlecan, nidogen and trophic factors. It allows 3D culture of many different cell types, however, it has limitations including batch-to-batch variability in quality, inability to mimic the mechanical properties of the basement membrane and inappropriateness to recapitulate other matrices of connective tissues due to the not-corresponding composition [525]. To overcome these limitations, more and more biomaterials are synthesized and applied which allow controlling their mechanical properties, the 3D architecture and biochemical composition.

Natural and synthetic polymers in different processing forms — mostly hydrogels and macroporous scaffolds or foams — are used for this purpose. Synthetic polymers often need to be further functionalized to allow efficient interactions with cells e.g. via adhesion [521], [522]. Amongst the natural polymers, proteins such as collagen/gelatin or serum albumins as well as carbohydrates including chitosan, alginate, dextran, hyaluronic acid or heparin are applied [474], [499], [510], [522], [525], [526]. More complex ECM mimics are provided by working with decellularized ECMs, derived either directly from bone or BM or from cell culture [522], [527], [528], [529], [530]. While they can preserve the natural composition and 3D architecture of the ECM, their composition is hardly ever exactly known, hampering the interpretation of results in terms of connecting observed cellular responses to a single ECM derived stimulus [525]. Besides, also inorganic compounds of bone including tricalciumphosphate or hydroxyapatite were applied [531]. All of these materials as well as scaffold-free approaches such as spheroid cultures have their advantages and disadvantages. Selection of an appropriate 3D culture system, and if needed ECM-mimicking biomaterial or scaffold, depends on the requirements of the intended application (see Table 2).

**Table 2:** Comparison of four ECM-mimicking strategies. The effectiveness of the different strategies was rated concerning the criteria named in the first column from poor (−) to very good (+++).

	*in vivo*/mouse	Matrix-free models	Bio-derived matrices	Tissue engineered hydrogels and scaffolds
Generation time	−	+++	++	+
Ease of manufacturing	−	+++	++	+
Reproducibility	−	++	+	+++
Complexity	+++	−	+	++
High throughput drug screening	−	+++	++	++
Low costs	−	+++	+	++
Biomimetic microenvironment	+++	−	+	++
Similarity to human ECM	++	−	++	++
Spatial heterogeneity	+++	+	+	+++

### 10.3 Toward synthetic, artificial stem cell niches

Besides mimicking the ECM with its biological and physical properties including its 3D architecture, cell–cell interactions via direct contacts and soluble factors are of utmost importance in the natural HSC niche. Accordingly, many studies that aim to recreate the niche combine 3D culture techniques with co-culture of HSCs with supporting cells. Bringing crucial parameters of the natural HSC niche — found in *in vitro* and *in vivo* studies — in this way together into one system is the basis for creating so-called artificial HSC niches. The number of studies trying to recreate the HSC niche by mimicking these factors and parameters to a certain extent is constantly rising and for a complete overview the reader is referred to other excellent recent reviews focusing on this topic [510], [532]. Many different approaches including different cell types, scaffolds, 3D culture techniques and bioreactors were taken, and, so far, none of these approaches became dominant in the field [532]. Currently, these artificial HSC niches are mainly used for HSC multiplication for potential future application of such expanded cells in cellular therapies. However, employing these models also for fundamental studies on the healthy and diseased human HSC niche as well as for disease modeling and as platforms for drug testing is promising and might have implications for research, drug development and personalized medicine in the future.

### 10.4 Artificial stem cell niches for disease modeling and drug development

To date, most studies on artificial HSC niches aimed at recreating the healthy BM and evaluating the potential of these systems for HSPC multiplication [510], [533]. To a lesser extent also production of mature cell types was investigated in such systems for red blood cell or platelet production [534], [535]. Surprisingly little attention has been paid to the application of such *in vitro* systems for evaluating hematotoxicity, although this is an important parameter in drug development and application. However, artificial niches cannot only help to assess potential side effects and toxicities of drugs, they can also be utilized to predict the effectivity of drug treatment in the BM, by modelling the BM in certain pathologies, including malignant or infectious diseases.

#### 10.4.1 Systems to evaluate drug induced BM toxicities

The hematopoietic system produces billions of blood cells on a daily basis. The accordingly high proliferation rates as well as the intimate connection of HSC niches with blood vessels make the hematopoietic system very sensitive to the treatment with drugs including chemotherapeutics [518], [536], [537]. The resulting hematotoxic effects can lead to anemia, neutropenia, thrombopenia or pancytopenia causing severe symptoms from poor oxygen saturation due to lack of erythrocytes via the vulnerability to infections because of missing or reduced numbers of immune cells to blood clotting deficiencies as platelet counts drop [518]. These symptoms or combinations of them can yield life-threatening conditions.

Due to the susceptibility of the blood-forming system in the BM to many drugs and the severity of occurring hematotoxic effects, there is an urgent need for pre-clinical screening tools that enable reliable and robust prediction of BM toxicities. Recreating the human HSC niche might be of particular importance in these approaches in order to enhance the predictive power of such systems in comparison to animal experiments, as species-related differences are particularly present in the hematopoietic systems [455].

Currently, colony-forming-unit (CFU) assays are used to predict hematotoxicity *in vitro*. This approach was validated by the European Center for the Validation of Alternative Methods [538]. CFU assays allow the retrospective enumeration of different hematopoietic progenitors in a cell population by culturing HSPCs in cytokine-supplemented semi-solid media. Different progenitors lead to the formation of different kinds of colonies characteristic for different blood cell lineages. The following visual inspection of the arisen colonies by light microscopy allows the enumeration of the different types of colonies and, thereby, the analysis of effects of an added drug on different blood cell progenitors. Despite their advantages, CFU-assays suffer from severe drawbacks including (i) the subjectivity in the characterization and enumeration of colonies hampering standardization, (ii) the lack of possibilities to detect secondary toxicities or effects in the hematopoietic microenvironment, (iii) the difficulty to achieve mechanistic insights and (iv) the impossibility to reconcile chronic treatments [518]. Artificial BM analogs have the potential to overcome these limitations.

Using a macroporous scaffold mimicking trabecular bone to co-culture HSPCs and BM-derived MSCs in a perfusion bioreactor, we were able to reconcile processes in the BM in healthy steady-state conditions — namely balanced HSPC maintenance and differentiation — and in activated alarm situations, which lead to enhanced HSPC differentiation to accommodate the need for elevated blood cell numbers under such conditions. Challenging this system with 5-fluorouracil, a chemotherapeutic agent well known to be hematotoxic, showed that the myelotoxicity of this drug was heavily underestimated in 2D cultures and that the type of hematopoietic cells mainly affected by the drug depended on the mimicked physiological state of the BM [497]. Using magnetic macroporous hydrogels with contactless motion control of the gels inducing perfusion within materials, is one potential way to enhance the throughput of such systems for screening assays [498].

Bourgine et al. developed a BM analog that consists of a bone-like ceramic scaffold and human stromal and osteoblastic cells, the ECM deposited by them and CD34^+^ HSPCs. The scaffold is integrated in a perfusion bioreactor. The resulting tissue supported HSPC maintenance and differentiation as well as recreation of a complex ECM containing collagen type I and IV, fibronectin and osteocalcin. Treatment of the construct with bleomycin yielded a diminished capacity of MSCs to support HSC quiescence accompanied by enhanced HSC proliferation [170].

The most advanced artificial HSC niches for drug testing are so-called BM-on-chip devices. Organs-on-chips allow principally for high throughput drug screening [539]. So far, only few BM-on-a-chip devices have been described. Torisawa and colleagues were the first to present such a system. In 2014 they described a mouse-derived *ex vivo* BM-on-chip and demonstrated its suitability to assess physiological effects of gamma-irradiation, G-CSF and bactericidal agents [540], [541]. A few years later Sieber et al. published the first human BM-on-chip. The system included a 3D zirconium oxide scaffold coated with hydroxyapatite that was seeded with human HSPCs and MSCs. The device allowed long-term survival of HSPCs in culture with a population that was stable for 28 days [542]. Recently, Chou et al. presented a BM-on-chip, in which HSPC maintenance and differentiation to mature blood cells was balanced. The device consisted of two channels. The vascular channel was made from polydimethylsiloxane (PDMS) and lined with endothelial cells, which was used to perfuse the hematopoietic channel via a porous membrane. The hematopoietic channel was filled with a MSC- and HSPC-laden fibrin gel. This system supported the maintenance and differentiation to myeloid blood cell lineages for several weeks. Its exposure to chemotherapeutic drugs recapitulated the BM toxicities observed *in vivo* including myelotoxicity. The system was also suitable to mimic diseases as demonstrated by using cells from patients with Shwachman-Diamond-syndrome [543]. Furthermore, the chip was also integrated into a multi-organ-on-chip device together with models for kidney and liver for successful pharmacokinetic modeling, which was exemplarily shown for cisplatin [544].

#### 10.4.2 Artificial niches for testing efficacy of drugs in BM-associated diseases

Artificial stem cell niches that mimic the BM under pathological conditions focus mainly on malignant diseases of the hematopoietic system including leukemia and multiple myeloma and bone metastasis, which are described in the following. However, also infectious diseases have been modelled, e.g. a model was developed that allowed to assess the effects of implant-associated osteomyelitis on the hematopoietic system [499].

Mimicking bone metastasis is of high relevance because of the high number of affected patients and because this stage represents a point-of-no-return in cancer progression. Particularly for breast cancer many models have been developed. Similar to *in vitro* models of healthy BM, also bone metastasis models evolved from single cultures of cancer cell lines, via co-cultures in 2D up to complex 3D environments seeded with multiple cell types. However, most of the published models are still in developmental stages and were rarely used to obtain mechanistic insights into bone metastasis but rather concentrated on validation of the models. The described models allow investigating early steps of bone metastasis, extravasation processes and colonization of bone including invasion and interaction with the new microenvironment which can go along with induction of dormancy or growth of the immigrating tumor cells [545], [546]. At the same time, the microenvironment also affects the sensitivity of tumor cells to chemotherapeutics as shown e.g. by co-cultures with MSCs in 3D structures [547], [548].

Leukemia is a malignant disease of the hematopoietic system, yielding finally its flooding with leukemic blasts to the expense of healthy hematopoiesis [549]. Similar to studies aiming at mimicking the healthy niche, mimicking the 3D ECM and support by stromal cells appeared to be crucial for reconciling the leukemic BM. However, already relatively simple studies culturing leukemic cell lines in 3D indicated that the tumor microenvironment can lead to increased drug resistance as shown for example in experiments using Jurkat cells in collagen type I-coated polycaprolactone scaffolds and exposing them to cytarabine and daunorubicine [550]. The next level of complexity can be added by introducing supporting cells such as osteoblasts or MSCs into the systems and/or by working with primary leukemic cells. Such studies showed the supportive character of the microenvironment for leukemic cells which leads to enhanced resistance to the investigated chemotherapeutics [551], [552]. 3D co-culture systems were also applied to investigate the importance of the CXCL12/CXCR4 axis as a target during leukemia treatment. Disruption of this axis — that guides cells expressing the chemokine receptor CXCR4 via a CXCL12 gradient into BM niches — by pretreatment with inhibitors enhanced leukemic cell migration and the sensitivity of leukemic cells to applied drugs [531], [553], [554]. The protective effect of the niche and particularly its ECM for leukemic cells during therapy was shown by inhibiting the adhesive interaction between leukemic cells and osteopontin in combination with a tyrosine kinase inhibitor in a 3D co-culture with osteoblasts, which led to an enhanced sensitivity to drugs [531], [555].

Triculture models, incorporating leukemic cells together with two further cell types showed promising results as artificial leukemic niche models. Bruce *et al.* employed a triculture system to model the endosteal BM microenvironment during leukemia, by combining a 3D collagen matrix with osteoblasts, MSCs and a human leukemic cell line. They found that the sensitivity of leukemic cells to chemotherapeutics was decreased in 3D systems when compared to 2D, indicating enhanced chemoresistance in 3D. They concluded that this effect was most likely caused by differences in cell–ECM interactions in 2D and 3D that occur via differences in integrin localization in the cells’ membrane, their activation and downstream signal transduction. Furthermore, collagen matrices might act as a barrier for drugs that thwart their diffusion in the matrix and thus diminish the concentration and thereby efficacy of the drug at the target site [556]. Bray et al. presented in 2017 an *ex vivo* triculture model that mimicked the interaction of leukemic cells with the vascular niche. They equipped an MMP-sensitive starPEG-heparin hydrogel with adhesive ligands (RGD) and proangiogenic factors and used this system to co-culture leukemic cell lines or primary patient-derived leukemic cells together with endothelial cells and MSCs. They showed that the drug resistance of leukemic cells was greater in 3D and in vascular co-cultures than in 2D suspension cultures and that their model was suitable for personalized analyses of drug responses of patient’s cells [557].

Similarly, it was found for multiple myeloma that 3D cultures with support by stromal and endothelial cells improve multiple myeloma cell proliferation and increase their drug resistance [558], [559]. Furthermore, multiple myeloma models are already employed for tumor-on-chip models, because the cells are easily available and injectable into microfluidic BM-mimicking devices. These advanced systems allow to study the drug response in a dynamic context [560].

All in all, it appears that 3D culture and adhesion of leukemic cells to a stromal niche enhance their survival rates during drug treatment, possibly by induction of a phenomenon called cell adhesion mediated drug resistance (CAM-DR), by promoting leukemic cells to enter a quiescent state in which they are protected from drugs that act on quickly proliferating cells and by increasing hypoxic regions that might also contribute to chemoresistance [525]. Thus, not only the malignant cells themselves but also their tumor microenvironments including the ECM therein are potential targets for molecular therapies.

In conclusion, all of the presented studies show that engineered 3D models of healthy and diseased BM, mimicking the ECM with the necessary degree of complexity from biochemical composition to 3D architecture and cell–cell interactions are necessary to develop predictive *in vitro* models to assess toxicity and efficacy of drugs.

## 11 Conclusion and perspectives

If compared to studies on cellular components, the ECM of the HSC niche is still largely underexplored. While early studies described the expression profiles of different ECM molecules in BM and investigated the effects of them on isolated HSPCs or hematopoietic cell lines, more recent studies show a functional involvement of ECM molecules in HSC niche biology from cell adhesion and anchorage via HSC migration, motility and mobilization all the way to storage, release and diffusion of soluble molecules involved in HSC regulation. This multitude of functions is exerted by the complex composition of different ECM molecules in BM leading to defined biochemical properties as well as the biophysical characteristics of the resulting matrix. All in all, it is evident that the ECM is a crucial part of the HSC niche that is indispensable for proper niche function. Therefore, mimicking ECM with the required degree of complexity is also inevitable for approaches aiming at applications such as HSC *in vitro* expansion or targeted differentiation for cellular therapies or *in vitro* models of BM for drug testing or fundamental research of the healthy or diseased BM. While currently many of these studies still rely on natural molecules, future research will aim at fully defined and synthetic ECMs as culture substrates to enable full control of the physical and chemical properties and to comply with Good Manufacturing Practices (GMP) thus enabling application of the matrices in clinical trials.

## Funding

This project has received funding from the European Research Council (ERC) under the European Union’s Horizon 2020 research and innovation programme (grant agreement No 757490). This work has been carried out within the framework of the SMART BIOTECS alliance between the Technische Universität Braunschweig and the Leibniz Universität Hannover. This initiative is supported by the Ministry of Science and Culture (MWK) of Lower Saxony, Germany.

## Declaration of Competing Interest

The authors declare that they have no known competing financial interests or personal relationships that could have appeared to influence the work reported in this paper.
